# Identification of immune-related subtypes of colorectal cancer to improve antitumor immunotherapy

**DOI:** 10.1038/s41598-021-98966-x

**Published:** 2021-09-30

**Authors:** Xiaobo Zheng, Yong Gao, Chune Yu, Guiquan Fan, Pengwu Li, Ming Zhang, Jing Yu, Mingqing Xu

**Affiliations:** 1 Department of Liver Surgery, West China Hospital, Sichuan University, Chengdu, 610041 Sichuan China; 2 Department of Gastroenterology, Second Affiliated Hospital, Army Medical University, Chongqing, 400037 China; 3 Laboratory of Tumor Targeted and Immune Therapy, State Key Laboratory of Biotherapy, Clinical Research Center for Breast, West China Hospital, Sichuan University, Chengdu, 610041 Sichuan China; 4 Department of General Surgery, First People’s Hospital of Liangshan Yi Autonomous Prefecture, Liangshan, 615000 Sichuan China; 5 Department of Hepatobiliary Surgery, Chongzhou People’s Hospital, Chengdu, 611200 Sichuan China; 6 Department of General Surgery, Mianzhu Hospital of West China Hospital, Sichuan University, Mianzhu, 618200 Sichuan China; 7 Department of Hepatopancreatobiliary Surgery, Meishan City People’s Hospital, Meishan Hospital of West China Hospital, Sichuan University, Meishan, 610041 Sichuan China

**Keywords:** Tumour immunology, Cancer microenvironment

## Abstract

Immunotherapy involving immune checkpoint inhibitors (ICIs) for enhancing immune system activation is promising for tumor management. However, the patients’ responses to ICIs are different. Here, we applied a non-negative matrix factorization algorithm to establish a robust immune molecular classification system for colorectal cancer (CRC). We obtained data of 1503 CRC patients (training cohort: 488 from The Cancer Genome Atlas; validation cohort: 1015 from the Gene Expression Omnibus). In the training cohort, 42.8% of patients who exhibited significantly higher immunocyte infiltration and enrichment of immune response-associated signatures were subdivided into immune classes. Within the immune class, 53.1% of patients were associated with a worse overall prognosis and belonged to the immune-suppressed subclass, characterized by the activation of stroma-related signatures, genes, immune-suppressive cells, and signaling. The remaining immune class patients belonged to the immune-activated subclass, which was associated with a better prognosis and response to anti-PD-1 therapy. Immune-related subtypes were associated with different copy number alterations, tumor-infiltrating lymphocyte enrichment, PD-1/PD-L1 expression, mutation landscape, and cancer stemness. These results were validated in patients with microsatellite instable CRC. We described a novel immune-related class of CRC, which may be used for selecting candidate patients with CRC for immunotherapy and tailoring optimal immunotherapeutic treatment.

## Introduction

Colorectal cancer (CRC) is the third most common malignancy reported worldwide and is the second leading cause of cancer-related deaths^1,2^. Early screening, surgical procedure advancements, and multidisciplinary systemic therapies have led to a decline in CRC mortality rates, and have helped achieve a 5-year survival rate of 90% in early-stage patients^3–5^. However, the 5-year survival rate is less than 15% among patients diagnosed with metastasis, and 25% of patients newly diagnosed with CRC are expected to present with metastases at the time of diagnosis^6,7^. Moreover, the incidence of early-onset CRC, which is defined as CRC occurring in individuals < 50 years of age, continues to increase considerably^8,9^. CRC is highly heterogeneous, resulting in varied prognoses even among patients exhibiting the same tumor stage, which poses challenges in the clinical management of the patients. The heterogeneous nature of CRC may be associated with differences in genetic mutations, the tumor microenvironment (TME), gut microbiota profile, and metabolism characteristics^10–12^. Therefore, the classification of CRC at the molecular level is urgently needed to improve risk stratification and adopt precision therapies for CRC prevention and management.

Tumor evolution is a consequence of complex interactions between tumor cells and the TME, including immunocytes and stromal cells. Immunotherapy has transformed the traditional paradigm of clinical cancer therapy and exhibits potent anti-tumor efficacy in various cancer types^13–16^. Application of monoclonal antibodies to inhibit immune checkpoints has resulted in the achievement of promising and durable responses in multiple cancers^17–20^. However, immune checkpoint inhibitors (ICIs) have shown effectiveness in only a few patients with CRC, and only those with the microsatellite instability (MSI) phenotype but not those with the microsatellite-stable (MSS) phenotype respond to PD-1 blockade (objective response rate [ORR], 40% vs. 0%)^21,22^. The MSI phenotype is represented by enrichment of tumor-infiltrating lymphocytes (TILs) and production of neoantigens, which facilitate immune activation. Moreover, accumulating evidence has shown that tumor mutation burden (TMB), PD-1/PD-L1 expression, and TILs correlate with ICI response^23–25^. However, a lack of thorough understanding of TME complexity has hindered the application of anti-tumor immune therapies. For example, immunotherapies that repress tumor-associated macrophages have been applied in the clinical setting, but minimal response has been observed with monotherapy^26^. Therefore, comprehensive profiling of the immune molecular characteristics of CRC is warranted.

Owing to technical advancements, several computational methods are available for dissecting tumor heterogeneity. Non-negative matrix factorization (NMF) analysis is a virtual microdissection approach that has been widely applied in numerous research fields, including image representation^27^, interpretation of multimodal data^28^, and omics analyses^29^. NMF can be used in the identification of cancer molecular subtypes based on gene expression profiles through the identification of exemplar genes^30–33^. In this study, we used NMF to virtually microdissect the molecular patterns that are closely associated with immune activation in CRC and validated the resulting molecular classification in three independent cohorts.

## Methods

### Patient selection

A total of 1503 patients with CRC, whose gene expression profiles and clinicopathological characteristics were available, were selected; the relevant data were obtained from public databases. The Cancer Genome Atlas-Colon Cancer (TCGA-COAD) and The Cancer Genome Atlas-Rectal Cancer (TCGA-READ) datasets were downloaded from UCSC XENA (http://xena.ucsc.edu/) and used as the training cohort. Only patients diagnosed with adenocarcinoma (n = 488) were selected for the subsequent analysis. Three additional CRC datasets with the required clinical information were obtained from the Gene Expression Omnibus (GEO) database (http://www.ncbi.nlm.nih.gov/geo/) and used as an external validation cohort. The three cohorts—GSE39582, GSE14333, and GSE17538—comprised 1015 patients.

### Extraction of immune expression pattern

We used the NMF package (v0.23.0) to perform microdissection of the mRNA expression profiles in the TCGA-COAD and TCGA-READ datasets. After testing, it was found that K = 10 was the number of factors that could be applied for good segmentation of TCGA data. To extract data on immune-related NMF factors, ESTIMATE was used to analyze the immune enrichment score in TCGA samples. Statistical analysis revealed that among all NMF factor groups, patients whose data were decomposed into the seventh factor had a higher immune enrichment score; hence, the seventh factor was defined as an “immune factor.”

According to the difference between the load value of the seventh factor and the maximum load of other factors, all genes were sorted from high to low, and the genes with the top 150 weights were considered to be the key factors distinguishing immune and non-immune classes. This classification was achieved through the utilization of the NMF Consensus command of the NMF package with top 150 genes, which was further refined using the multidimensional scaling (MDS) random forest method of the randomForest (v4.6-16) package. The clusterProfiler (v3.14.3) package was used to analyze the function of the 150 immune-related genes.

### Further classification of immune classes

Nearest template prediction (NTP) (CMScaller_0.99.2 package) and the single-sample Gene Set Enrichment Analysis (ssGSEA) method in the Gene Set Variation Analysis (GSVA) (v1.34.0) package were used to evaluate gene expression signatures and enrichment of molecular pathways that represented different inflammatory states or immune cells. Through NTP of stroma activation, the immune class data were further bifurcated into immune-suppressed and immune-activated subtypes. DESeq2 software was used to select genes that were significantly differentially expressed between the immune and non-immune groups with *p*_adj_ < 0.05 and absolute value of log2 fold change (FC) > 1. To determine the gene sets and pathways enriched in the immune and non-immune groups, the clusterProfiler (v3.14.3) package was used to conduct Kyoto Encyclopedia of Genes and Genomes and Gene Ontology pathway functional enrichment analysis of the differentially expressed genes (DEGs); simultaneously, all genes were enriched through GSEA using the fgsea (v1.12.0) package.

### Validation of immune subtypes in the validation cohort

We screened the top 150 dysregulated genes between the immune and non-immune classes. Similar to the training cohort, the NMF and the ESITIMAT algorithms were adopted to separate the validation cohort samples into immune and non-immune classes based on the expression of the selected 150 genes. Furthermore, NTP was used to further divide the immune group into immune-suppressed and immune-activated subtypes.

### Prediction of response to immunotherapy

To predict the responses to ICI therapy in patients with CRC, tumor immune dysfunction and exclusion (TIDE) and SubMap analysis (GenePattern module “SubMap”) were used. SubMap is an algorithm used to assess similarities in gene expression between previously defined immunophenotypes and responders or non-responders to anti-PD-1 treatments in the MD Anderson melanoma cohort.

### Correlation of immune class with copy number alterations, mRNA stemness index, neoantigens, TILs, TMB, and mutational genes

Copy number alterations (CNAs) were calculated using GISTIC2.0 from GDAC Firehose (https://gdac.broadinstitute.org). A statistical comparison was then conducted to determine the difference between the immune and non-immune groups in amplification or deletion events at the arm and focal levels. Previously, Rooney et al*.* calculated the neoantigen number of TCGA tumors, from which the neoantigen number of TCGA-COAD and TCGA-READ could be obtained^34^. Furthermore, Saltz et al*.* estimated the abundance of TILs based on the images of hematoxylin and eosin (H&E)-stained sections of 13 TCGA tumor types, which included data on CRC^35^. Mutation data were collected from TCGA (https://tcga-data.nci.nih.gov), and the TMB was evaluated using the maftools (v2.6.05) package. To obtain data on the significantly mutated cancer genes (*p* < 0.01), we used the MutSigCV (v1.41) package to analyze the mutation data. Independent tests were then performed to further determine the mutations with significant differences among the different groups (*p* < 0.05). Finally, the mutation landscape oncoprint was generated using maftools. The mRNA stemness index (mRNAsi) was analyzed through one-class logistic regression, which can help predict stemness in poorly differentiated tumors.

### Statistical analysis

Unless otherwise noted, all analyses were conducted using R software (v3.6.1), and the significance level was set at *p* < 0.05. Normally distributed data were analyzed using the Student’s *t*-test and analysis of variance, whereas non-normally distributed data were analyzed using the Wilcoxon and Kruskal–Wallis tests. For the analysis of categorical variables, the Fisher's exact and Pearson's chi-square tests were used (ns *p* > 0.05, *p < 0.05, **p < 0.01, ***p < 0.001, or ****p < 0.0001). Kaplan–Meier analysis and the log-rank test were conducted to compare patients’ survival between different immunophenotypes.

## Results

### Identification of a novel immune-related subtype of CRC

**Figure 1:**
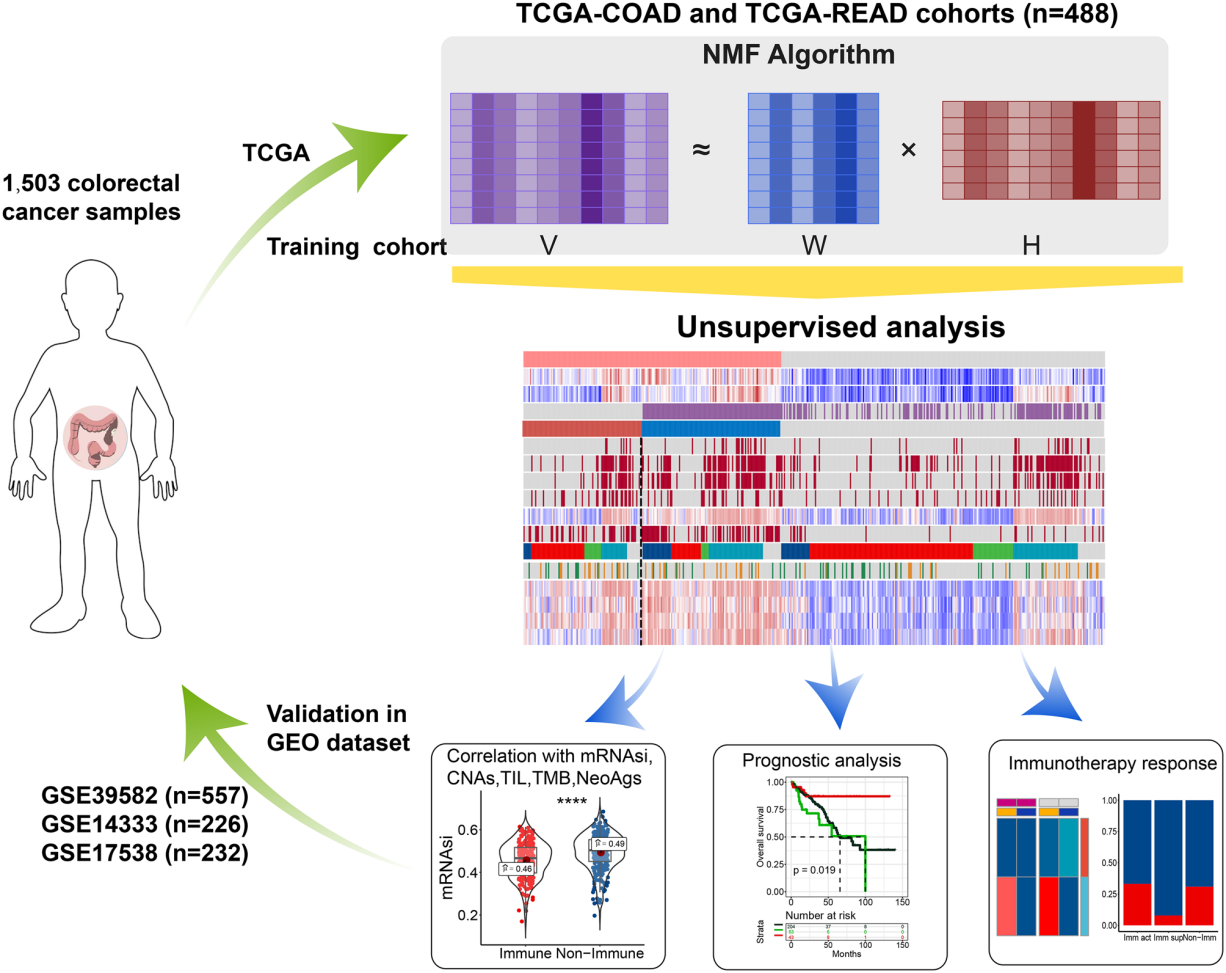
Flow chart of the analyses conducted in this study.

**Figure 2:**
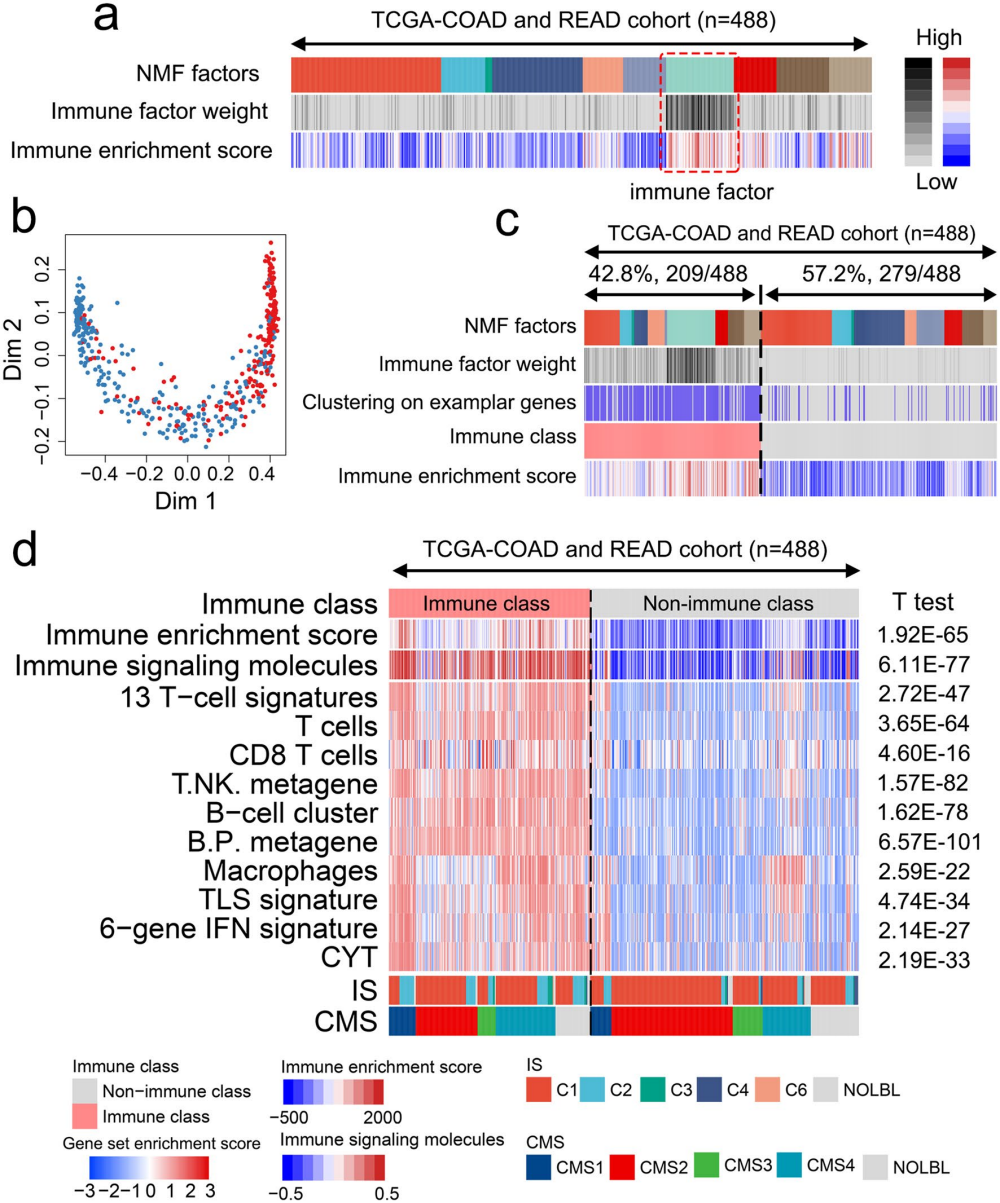
Identification of novel immune-related classes of colorectal cancer using the non-negative matrix factorization (NMF) approach. (**a**) Integration of NMF and ESTIMATE algorithms revealed the seventh factor enriched in most patients with high immune enrichment scores. (**b**) Data on the immune and non-immune classes were refined through Multidimensional Scaling random forest analysis through the top 150 exemplar genes. (**c**) Heatmap showing the distribution of patients with different NMF factors, immune factor weight, clustering based on exemplar genes, immunophenotypes, and immune enrichment score. (**d**) Heatmap showing the gene set variation analysis scores of immune-related signatures reported previously between the immune and non-immune classes. The Student’s *t*-test was used to statistically analyze enrichment scores between the immune and non-immune classes. *TLS* tertiary lymphoid structure, *CYT* cytolytic activity score, *CMSs* consensus molecular subtypes, *ISs* immune subtypes.

To establish an immune-associated molecular classification of CRC, we selected 488 patients from TCGA for a training cohort and 1015 patients from the GEO for a validation cohort, to dissect gene expression profiles using the NMF algorithm (Fig. 1). The omics sequencing data and complete clinical information of the patients included in this study are presented in Table 1. We first used the NMF algorithm to conduct a virtual microdissection of distinct gene expression patterns in the training cohort. Upon integration with the immune enrichment score^36^ generated using the ESTIMATE algorithm, the seventh of the 10 expression patterns (NMF factors) was found to be enriched in patients with high immune enrichment scores and exhibited a significantly higher immune enrichment score average than most of the remaining patterns (Fig. 2a and Supplementary Fig. S1). Therefore, this pattern was considered as an immune factor. The top 150 weighted genes in the immune factor were defined as exemplar genes, which represented the immune factor expression pattern. We performed Gene Ontology enrichment analysis for the exemplar genes and observed that immune activation-associated pathways, such as humoral immune response, lymphocyte-mediated immunity, complement activation, and antigen binding, were significantly enriched (Supplementary Fig. S1), indicating the immune-related functions and signaling of the immune factor.

**Table 1:** Summary of the clinicopathological characteristics of four independent colorectal cancer datasets.

	TCGA	GSE39582	GSE14333	GSE17538
Overall	488	557	226	232
**Age**				
≥ 70	286	319	129	146
< 70	202	237	97	86
**Sex**				
Male	267	307	120	122
Female	221	250	106	110
**Tumor stage**				
Ta	18	–	–	–
T1	89	36	–	28
T2	175	260	–	72
T3	132	201	–	76
T4	74	60	–	56
**Primary site**				
Colon	364	557	–	232
Rectum	61	–	–	–
Rectosigmoid junction	61	–	–	–
Connective, subcutaneous, and other soft tissues	2	–	–	–
**DFS status**				
Disease-free	182	380	50	55
Recurred/progressed	78	177	176	145
**OS status**				
Living	227	–	–	139
Deceased	74	–	–	93

*DFS* disease-free survival, *OS* overall survival.

To further stratify immune-related CRC subtypes, consensus clustering based on the exemplar genes was conducted to obtain preliminary classes, and data for the classes were refined using an MDS random forest algorithm (Fig. 2b). This helped identify a novel immune molecular subtype that accounted for 42.8% of the cohort (209/488). This subtype was termed as the “immune class.” The remaining 57.2% (279/488) of the cohort was termed as the “non-immune class” (Fig. 2c). We characterized the function of the immune class using immune-related gene signatures derived from literature and databases (Supplementary Table S1) through GSVA analysis. The immune class showed significantly higher enrichment scores for immune-related signatures than the non-immune class, including immune infiltrating cell-related signatures (T cell, NK cell, B cell, macrophage), as well as cytotoxic effect-related signatures, such as tertiary lymphoid structure (TLS), interferon (IFN) signatures, and the cytolytic activity score (CYT) (all *p* < 0.05, Fig. 2d). Additionally, we compared the DEGs between the immune and non-immune classes and observed that these DEGs were significantly enriched in immune-activated signatures, such as cytokine-cytokine receptor interaction, Th17 cell differentiation, Th1 and Th2 cell differentiation, and antigen binding (Supplementary Fig. S2). We also compared the difference in enrichment signatures between immune and non-immune classes through GSEA, which revealed significantly upregulated immune response related-pathways in the immune class, such as the intestinal immune network for IgA production, cytokine-cytokine receptor interaction, inflammatory response, IFN gamma response, IFN alpha response, and tumor necrosis factor alpha signaling via NF-kB (Supplementary Fig. S2). These results indicated that the immune class identified depicted evident characteristics of immune activation.

To evaluate the accuracy of the NMF algorithm-generated immune molecular classification, we integrated features of the established immune class with previously reported CRC molecular features. Thorsson et al*.*^37^ established a method of pan-cancer immune classification (C1-C6) of solid tumors by conducting an extensive immunogenomic analysis of over 10,000 tumors derived from 33 distinct cancer types. We observed a significant enrichment of C2 (IFN-γ-dominant, 48/190 vs. 24/236, *p* < 0.01), and a significant decrease in C1 (wound healing, 130/190 vs. 201/236, *p* < 0.01), in the immune class compared with those in the non-immune class (Fig. 2d). Consensus molecular subtypes (CMSs; CMS1-CMS4) proposed by the CRC Subtyping Consortium are regarded as the most accurate classification for the stratification of CRC cases^38,39^. We observed a significant enrichment of CMS4 (mesenchymal subtype, 60/190 vs. 43/236, *p* < 0.01) and a significant decrease in CMS2 (canonical subtype, 62/190 vs. 120/236, *p* < 0.01) in the immune class compared with those in the non-immune class (Fig. 2d). Collectively, these results suggest that the immune-related classes identified in this study through the NMF analysis may be used to stratify CRC based on the immune molecular characteristics.

### Immune class is composed of immune-activated and immune-suppressed subtypes based on different tumor niches

**Figure 3:**
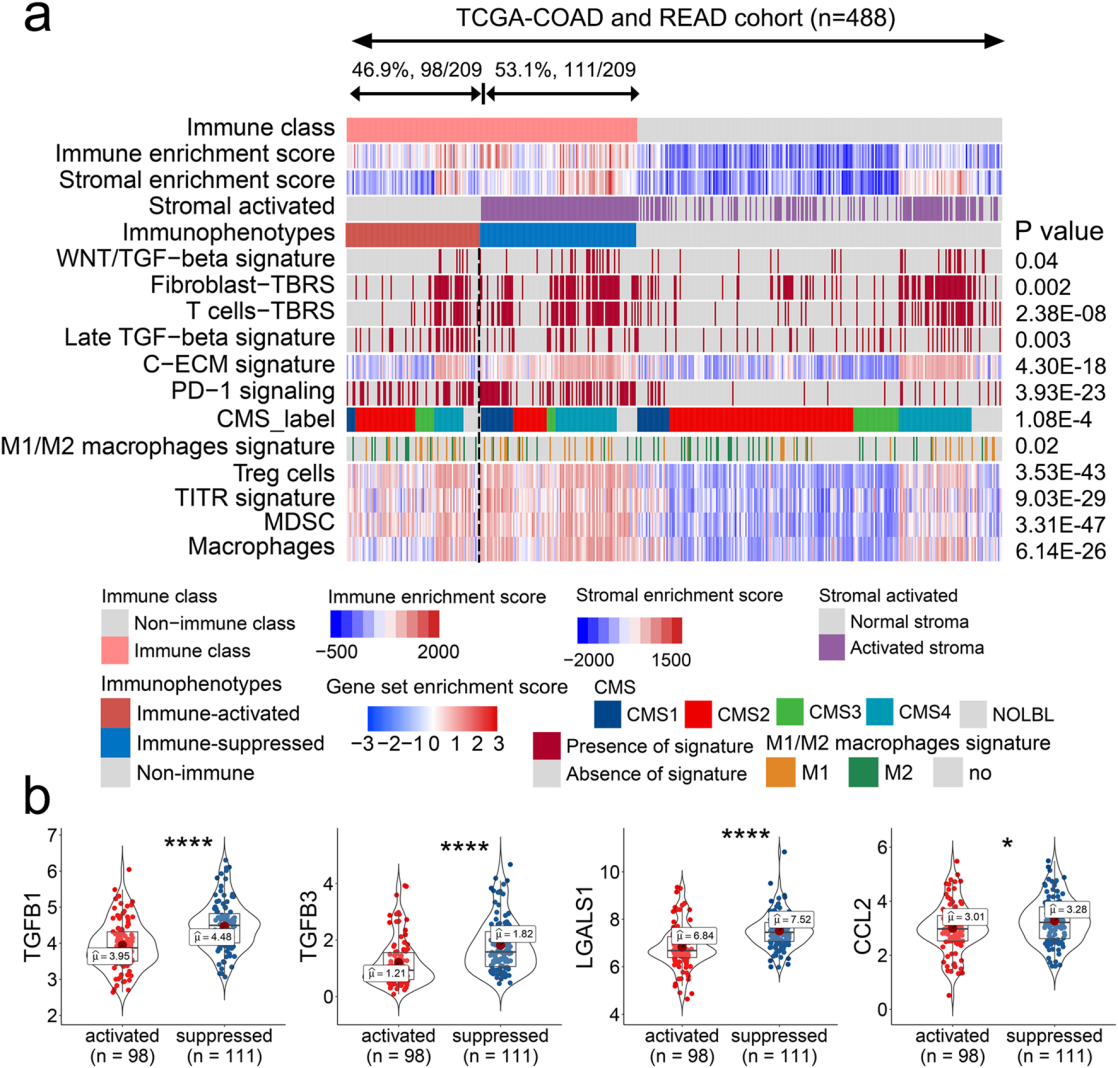
Identification of the immune-activated and immune-suppressed subclasses of colorectal cancer based on immunosuppressive signaling. (**a**) Heatmap showing the gene set variation analysis scores of immune-related signatures reported previously and the predicted result of the signature calculated by considering nearest template prediction between the immune and non-immune classes. (**b**) Expression of genes related to immune suppression between the immune-activated and immune-suppressed classes.

The TME varies in components of immunocytes and cytokines in the tumor niche, which confer anti- and pro-tumor activities during cancer progression. Moffitt et al. proposed stromal activation as a signature for the categorization of pancreatic ductal adenocarcinoma into normal and activated stromal subtypes, which was successfully applied to provide decision support and tailor therapies in the clinic^40^. To explore this component in the immune CRC class, we integrated NTP analysis by determining stromal activation signature. We identified 46.9% (98/209) of the patients in the immune class that lacked stromal enrichment, whereas the remaining 53.1% (111/209) had a relatively higher stromal enrichment scores (Fig. 3a). TGF-β is regarded as the pivotal regulator of immune suppression within the TME and has been reported to play roles in tumor immune escape and poor response to ICI immunotherapy^41,42^. Notably, we observed that multiple TGF-β signatures, such as WNT/TGF-β and TGF-β response signatures of fibroblasts (Fibroblast-TBRs), TGF-β response signatures of T cells (T cells-TBRs), late TGF-β, and extracellular matrix cytokines (C-ECM) were all significantly enriched in the stromal-activated class than those observed in the remainder of the immune class (all *p* < 0.05, Fig. 3a). We also observed that CMS4 subtypes and PD-1 signaling were higher enriched in the stromal-activated class than the remainder of the immune class (Fig. 3a). Therefore, we defined the stromal-activated class as the “immune-suppressed subclass” of the immune class. The remaining cases, which lacked the TGF-β and PD-1 signatures, were defined as the “immune-activated subclass.”

Cytotoxic T cells, Th1 cells, and IFN-γ potently eliminate tumor cells, whereas Treg cells, cancer-associated fibroblasts (CAFs), and myeloid-derived suppressor cells (MDSCs) are regarded as immune-suppressive cells^43,44^. Interestingly, we observed that immunosuppressive Treg cells, tumor-infiltrating Treg (TITR) cells, MDSCs, and macrophages signatures were significantly upregulated in the immune-suppressed subtype (all *p* < 0.05, Fig. 3a), which confirmed the suppressive status of this subclass. Additionally, we observed that the expression of immune-suppressive genes, such as TGFβ1, TGFβ3, LGALS1, and CCL2, was significantly higher in the immune-suppressed subclass than in the immune-activated subclass (Fig. 3b). Collectively, these results revealed that the immune class consisted of two distinct subtypes based on suppressive signaling.

### Reoccurrence of the three immune-related subtypes in other independent cohorts

**Figure 4:**
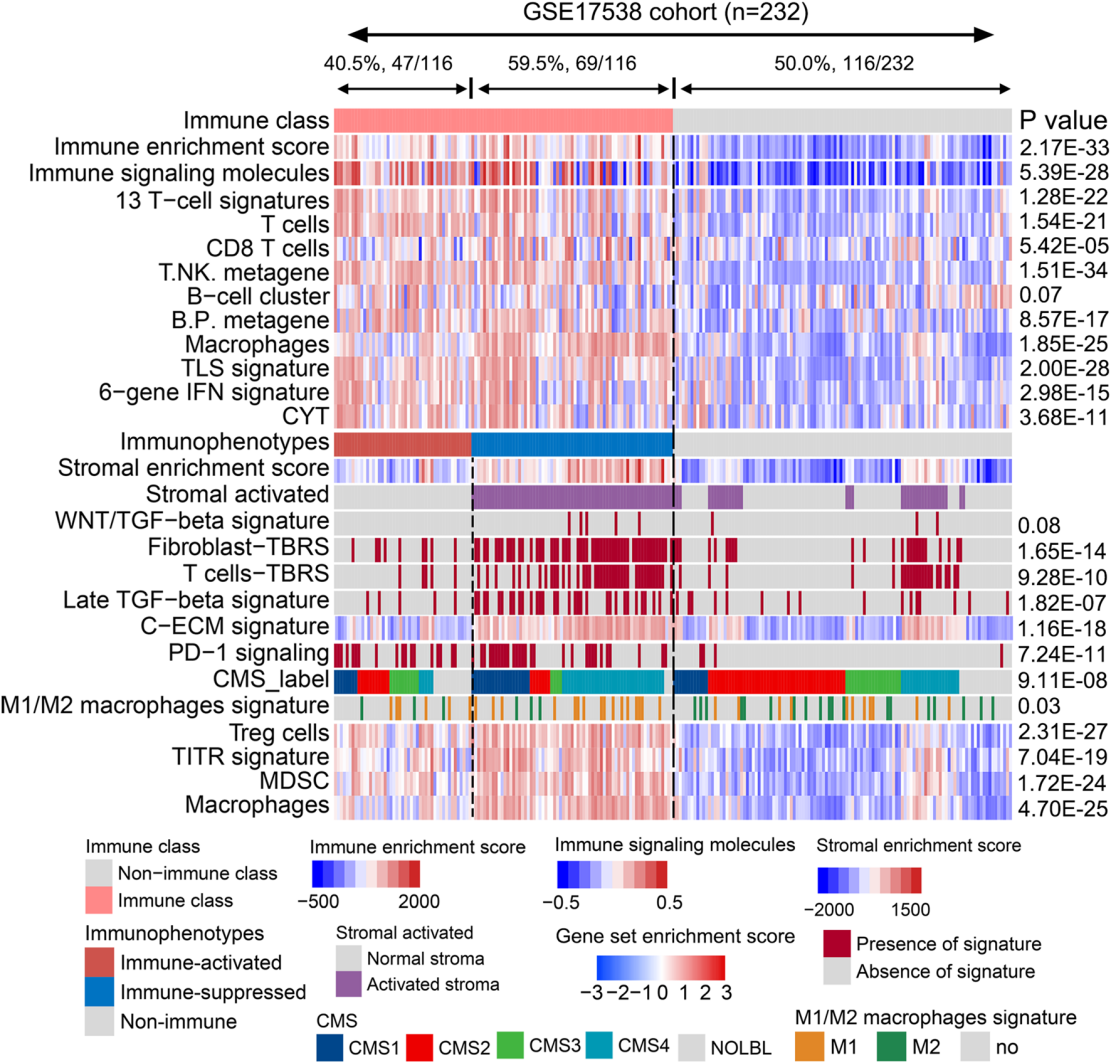
Validation of the immunophenotypes in the GSE17538 cohort.

To validate the recurrence of our established immune-related subtypes, we performed NMF analysis and activated stromal signature-based separation in three CRC cohorts (GSE17538, GSE14333, GSE39582, n = 1015; Table 1). The top 150 dysregulated genes identified between the immune and non-immune classes in the training cohort were considered as the immune classifiers. In the GSE17538 cohort, we identified 50.0% (116/232) of the patients who had a high immune enrichment score and belonged to the immune class, whereas the remaining 50.0% (116/232) were defined as the non-immune class. In the immune class, patients were further divided into immune-activated (40.5%, 47/116) and immune-suppressed (59.5%, 69/116) subclasses (Fig. 4). Similar to the results obtained for the training cohort, the immune class showed higher enrichment scores for the infiltrating immunocyte-related signatures, TLS, CYT, and IFN signatures, than the non-immune class. Accordingly, the immune-suppressed subclass displayed higher scores for stromal activation, TGF-β signatures, and immune-suppressive-associated signatures than the other two classifications. Similar results were also observed in the GSE14333 and GSE39582 cohorts. In GSE14333, 27.9% (63/226) patients were divided into the immune class, including 60.3% (38/63) of the patients grouped under the immune-suppressed subclass, and 39.7% (25/63) of the patients categorized under the immune-activated subclass (Supplementary Fig. S3). In GSE39582, 42.2% (235/557) of the patients were grouped under the non-immune class, whereas 50.9% (164/322) and 49.1% (158/322) of the remaining patients were grouped under the immune-activated and immune-suppressed subclasses, respectively (Supplementary Fig. S4). Genes related to immune suppression were upregulated in the immune-suppressed subclass in all three validation cohorts (Supplementary Fig. S5). Collectively, these results confirmed the stability and robustness of our immune-related CRC molecular classification system.

### Immune-related subtypes associated with prognosis outcome and response to ICIs

**Figure 5:**
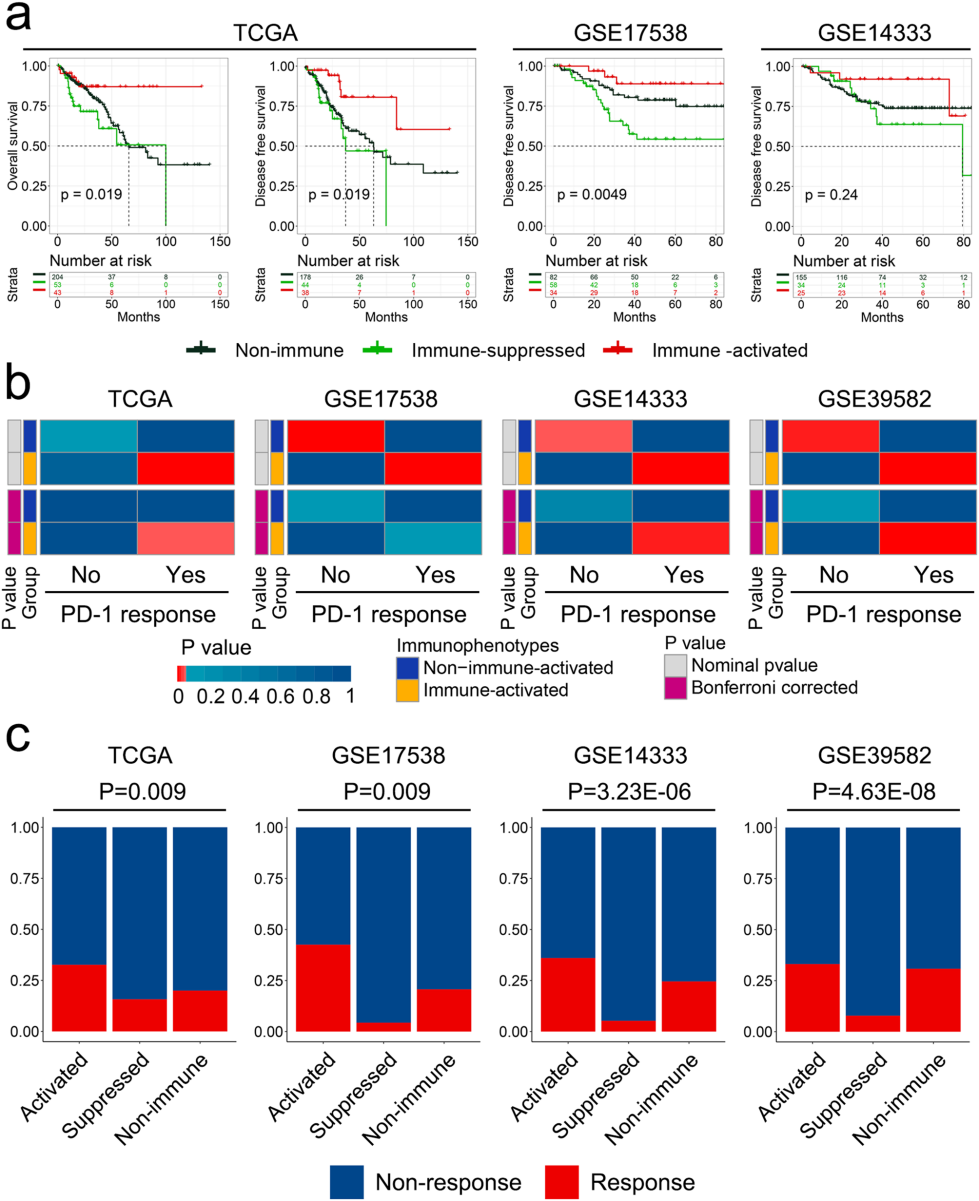
Immune-related molecular subtypes associated with prognosis and response to immune checkpoint inhibitor (ICI) therapy. (**a**) Kaplan–Meier analysis showing different overall and disease-free survival with three immunophenotypes in the training and validation cohorts. (**b**) Subclass mapping analysis indicates that patients in the immune-activated subclass show similarity with patients who respond to anti-PD-1 treatment. (**c**) Tumor immune dysfunction and exclusion analysis showing ICI response ratios among the three immunophenotypes.

To explore the clinical implications of this molecular classification, we first evaluated the different prognosis outcomes through survival analysis. The immune-activated subclass exhibited the best overall survival (OS) and disease-free survival (DFS) outcomes, the immune-suppressed subclass showed the poorest prognosis, and the non-immune class exhibited moderate prognosis outcomes in both training and validation cohorts (Fig. 5a). We performed the multivariate analysis to confirm the results of the survival evaluation. The results showed the immune-related classification is an independent factor of OS and DFS (Supplementary Table S2). We further explored the correlation between these groups and clinicopathological characteristics. No significant correlation was observed between the distinct immune subtypes and clinical features, including age, sex, tumor stage, and genetic mutation (Supplementary Table S3).

To assess the potential applicability of this immune-related molecular classification system in the prediction of response to ICI treatment, we used SubMap analysis to compare the gene expression pattern of our immunophenotypes with those obtained for patients with metastatic melanoma who were subjected to treatment with ICIs. Notably, patients with the immune-activated subclass exhibited a highly similar gene expression profile to melanoma patients who responded to anti-PD-1 immunotherapy in both training and validation cohorts (Fig. 5b). These results imply that patients in the immune-activated subclass may be potential candidates for receiving ICI immunotherapy. TIDE^45^, which is an effective computational method for predicting response to ICIs, was also used to evaluate the ability of our molecular classification to predict ICI response. We observed that the ratio of ICI response in the immune-activated subclass was higher than that in the other two groups, whereas the immune-suppressed subclass showed the worst ICI responses (Fig. 5c). Taken together, the immune-activated subclass exhibits the best prognostic outcomes and patients grouped under this subclass may benefit more from anti-PD-1 treatment.

### Relationship between immune-related subtypes and CNAs, TIL enrichment, PD-1/PD-L1 expression, and cancer stemness

**Figure 6:**
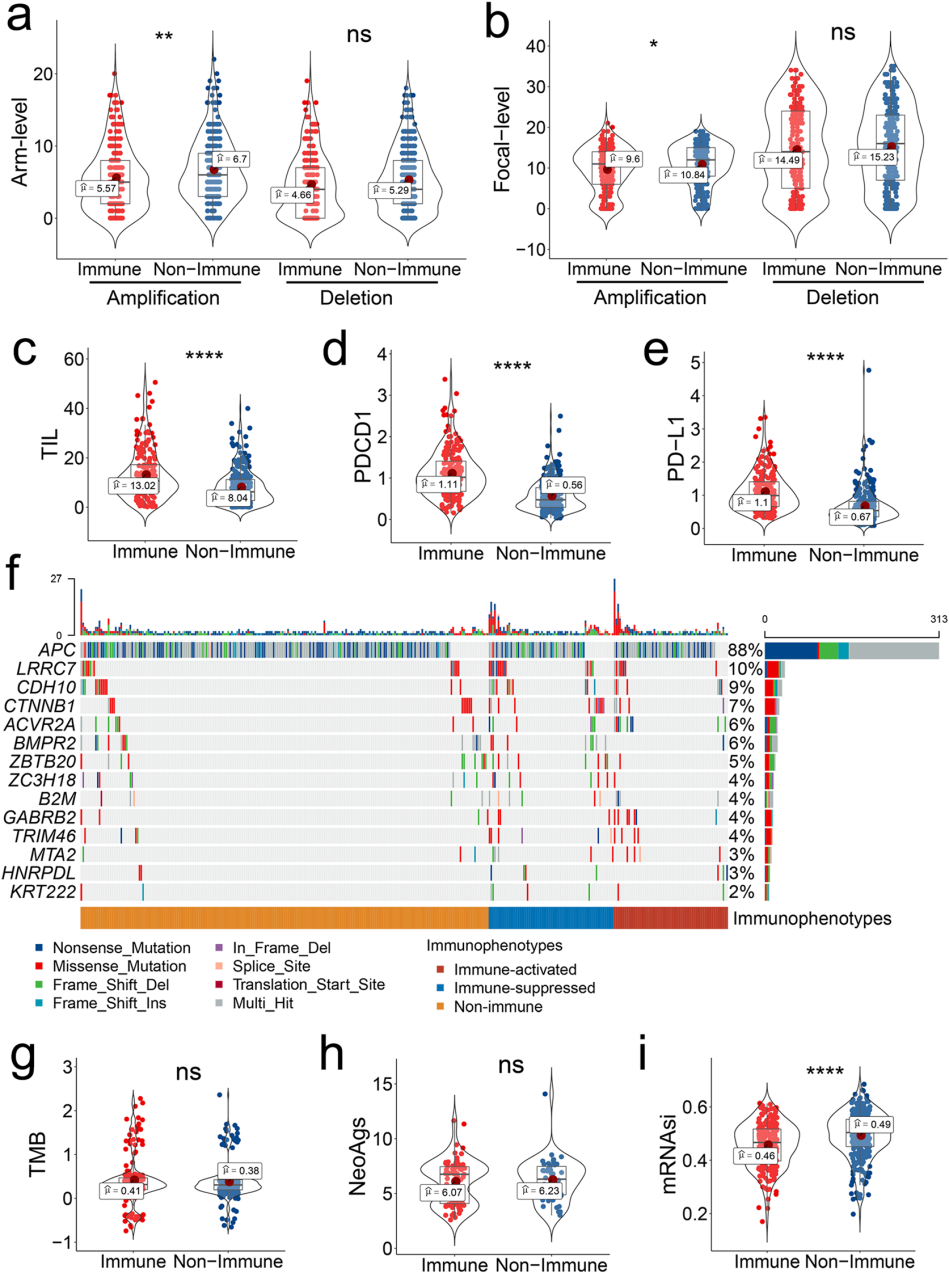
Correlation between immunophenotype and genetic characterization. (**a**, **b**) Copy number amplification and deletion at the arm and focal levels between the immune and non-immune classes. (**c**) Tumor-infiltrating lymphocyte count between the immune and non-immune classes. (**d**, **e**) PD-1/PD-L1 expression between the immune and non-immune classes. (**f**) Differentially mutated genes among the three immune subgroups. (**g**) Tumor mutant burden between the immune and non-immune classes. (**h**) Neoantigen content between the immune and non-immune classes. (**i**) Stemness difference represented by the mRNAsi signature between the immune and non-immune classes. Comparisons between groups were conducted using the Student’s *t*-test, whereas comparisons among three groups were conducted using the Kruskal–Wallis test, *t*-test, Student’s *t*-test; K–W test, Kruskal–Wallis test.

To illustrate the molecular mechanism underlying the different immune phenotypes, we analyzed the relationship between these immune-related subclasses and tumor molecular characteristics. CNAs are associated with distinct molecular characteristics, immunologic phenotypes, and clinical prognosis. Chromosomal instability is a common phenomenon in cancer and usually refers to arm and focal CNAs. Bassaganyas et al*.* found that tumors with high chromosomal instability exhibited characteristics of immune escape in hepatocellular carcinoma, whereas tumors with low burdens of arm CNAs displayed an immune-activated status, suggesting CNAs could be used to predict response to immunotherapies^46^. Consistent with the results, we observed that the immune class exhibited a significantly lower burden of copy number amplification than the non-immune class at both arm and focal levels, whereas there was no significant difference based on copy number deletion between the two classes (Fig. 6a, b). Notably, we found that the number of TILs was significantly upregulated in the immune class compared with those in the non-immune class (Fig. 6c). Moreover, we found that the expression of PD-1 and PD-L1 was significantly upregulated in the immune class compared with that in the non-immune class (Fig. 6d, e). Therefore, these well-accepted biomarkers used for predicting ICI response support the potential response of immune class to immunotherapy, suggesting a promising role for the immune-related molecular classification in guiding clinical immunotherapy.

Genetic characterization of tumor tissues has confirmed that high TMB and neoantigens, resulting from somatic non-synonymous coding mutations, may elevate tumor immunogenicity and increase susceptibility to immunotherapy^47^. We observed a different mutation profile among the three immune classifications via Mut-SigCV algorithm analysis (Fig. 6f). Notably, the mutation frequency of ACVR2A and GABRB in the immune-activated subclass was significantly higher than the immune-suppressed subclass and non-immune class (ACVR2A: 10.59% vs. 7.22% and 3.14%; GABRB: 8.24% vs. 3.61% and 1.57%; respectively). More mutations in LRRC7 and CDH10 were revealed in the immune-suppressed subclass than in other classes (LRRC7: 18.07% vs. 9.41% and 5.1%; CDH10: 14.46% vs. 8.24% and 4.71%; respectively). High TMB and neoantigen burden correlated with response to immunotherapy in various solid tumors, whereas this association was uncertain in MSS tumors^47^. Our study revealed that the TMB and neoantigen burdens were comparable between the immune and non-immune classes (Fig. 6g, h). Miranda et al*.*^48^ showed a negative correlation between stemness feature, represented by the mRNAsi signature, and the immune response. Accordingly, the mRNAsi signature expression was significantly lower in the immune class than in the non-immune class, implying that the immune class had reduced stemness and could be more responsive to immunotherapy (Fig. 6i). Collectively, these results indicate that the immune class possesses significantly higher TIL enrichment and PD-1/PD-L1 expression and lower copy number amplification and stemness, which may contribute to its immune response.

### Combination of immune-related subtypes and microsatellite status precisely guide immunotherapy strategy

**Figure 7:**
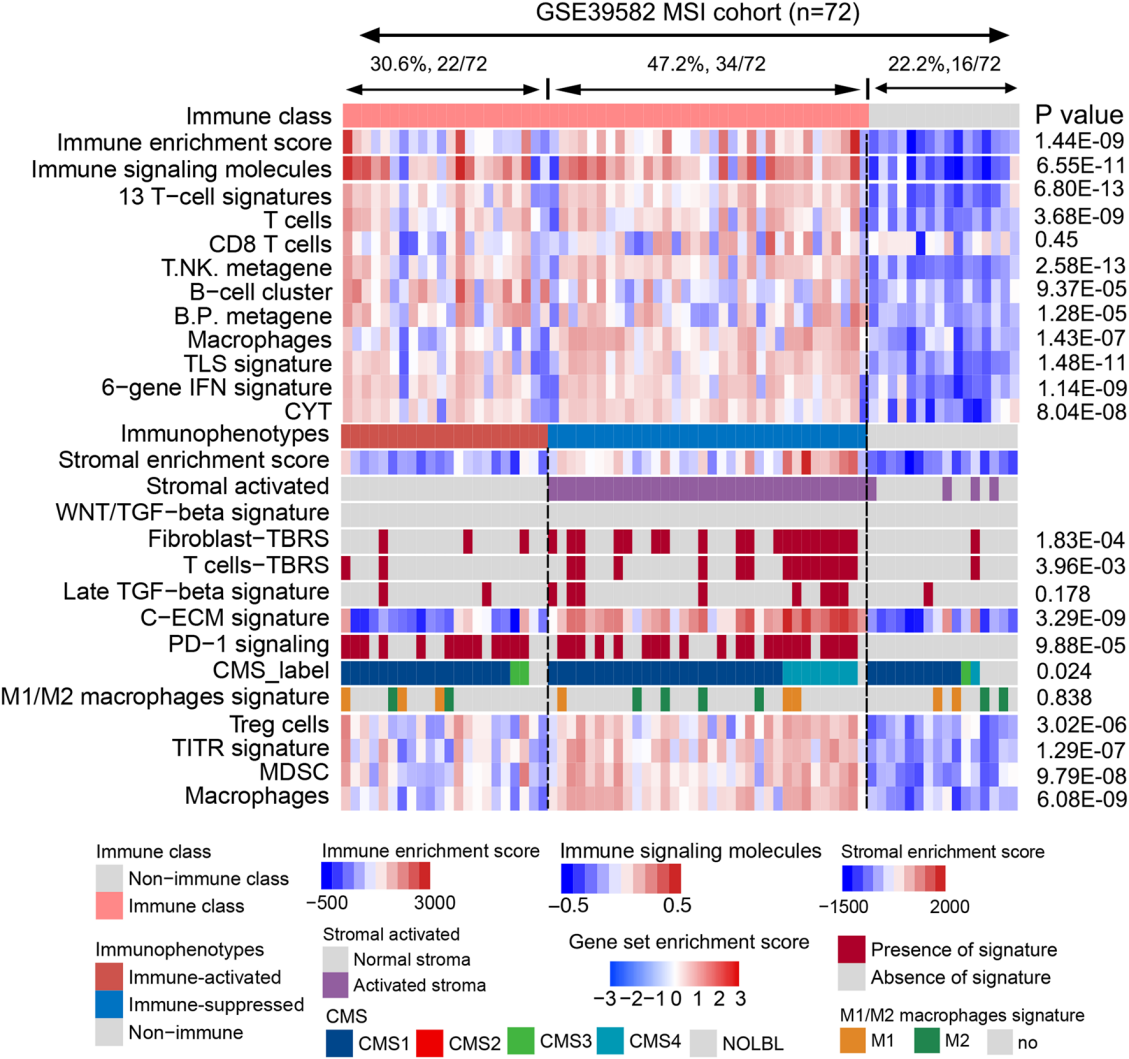
Validation of the immunophenotypes in patients with MSI CRC in the GSE39582 cohort.

Microsatellite status is an important factor involved in response to immunotherapy in CRC; patients with MSI CRC show a favorable outcome with immunotherapy. While only 40% patients with MSI CRC respond to ICIs, 60% patients with MSI CRC still do not respond to ICIs. Patients with MSI CRC who respond to ICIs and how to improve the response rate of patients with MSI CRC to ICIs are still unknown. We tries to integrate immune-related subclasses in patients with MSI CRC to explore heterogeneity in the effect of immunotherapy in patients with MSI CRC. We identified 72 patients with MSI from the GSE39582 datasets. Based on our established immune-related classification, these 72 patients were divided into non-immune, immune-suppressed, and immune-activated subclasses. Twenty-two (30.6%) patients were allocated to the immune-activated subclass, which exhibited higher immune cell infiltration and immune activation signaling, implying these patients were potential responders to ICI therapy (Fig. 7). Thirty-four (47.2%) patients were allocated to immune-suppressed subclass, which exhibited higher immune suppressive cells and immune suppressive signaling, such as TGF-β, PD-1, PD-L1, MDSC, macrophages, and Tregs (Fig. 7 and Supplementary Fig. S6). We recapitulated these results in the TCGA cohort; 51 patients with MSI CRC were identified, and 25 of them were allocated to the non-immune subclass, 20 patients to the immune-suppressed subclass, and 5 patients to the immune-activated subclass. Similarly, immune suppressive molecules and signaling were higher in the immune-suppressed subclass than in the non-immune and immune-activated subclasses, although no statistical significance was observed owing to small number of patients in the immune-activated subclass (Supplementary Fig. S7). Collectively, these results suggest that patients with MSI CRC allocated to the immune-activated subclass have a “hot” immune status. The tumor may be regressed by single ICI immunotherapy. In contrast, for patients in the immune-suppressed subclass, ICI therapy combined with a TGF-β inhibitor or an agent for the elimination of immune suppressive cells might improve efficacy. Our novel classification not only provides new insights and assists in identifying appropriate candidate patients with MSI CRC who will respond to ICIs, but also, through combination immune-related subtypes and MSI status, may help tailor optimal immunotherapeutic treatment and further improve clinical outcome of patients with CRC on immunotherapy.

## Discussion

Accumulating evidence has demonstrated the essential role of the TME in cancer progression^49^. However, the immune landscape in CRC remains poorly understood. A thorough investigation of different immune and stromal components in the TME may help understand complex tumor heterogeneity, develop more precise therapies, and refine current immunotherapeutic strategies. Particularly, establishment of an immune-related classification may provide data on more accurate subtypes of patients with CRC to help clinicians select more effective therapies. In this study, we presented a comprehensive immune-related classification of CRC based on the NMF algorithm according to CRC expression profiles. Using the top 150 exemplar genes of the immune pattern for consensus clustering, three immune-related molecular groups were identified, namely immune-activated, immune-suppressed, and non-immune. Molecular classification associated with different immune signaling pathways and immune cell types was established and validated in three independent cohorts. Notably, the immune-related classes correlated with patient prognosis and response to ICI therapy, and this finding might be used to tailor clinical immunotherapy strategies for patients with CRC.

The NMF algorithm, a multiplicative update algorithm, can be used to dissect substantial volumes of data using a limited number of components^50^. Emerging evidence has suggested NMF to be a promising method for classifying tumor molecular subtypes^51^. Using the NMF algorithm, we identified an immune pattern in the training cohort. Through consensus clustering of the exemplar genes of that immune pattern, CRC could be distinguished as immune and non-immune classes. Of the 488 patients in the training cohort, 42.8% were grouped under the immune class, which showed higher enrichment of infiltrating immune cells, cytolytic activity, and IFN signaling than the non-immune class. Effective immunotherapy is often limited by the immunosuppressive TME, which includes immunosuppressive cells and cytokines. Calon et al*.*^52^ revealed a pro-metastatic program induced upon secretion of IL11 by TGF-β-stimulated CAFs in the tumor niche. Accumulating evidence has suggested that the C-ECM level is upregulated by activated CAFs to trigger the recruitment of immunosuppressive cells^53^. To investigate the different components in the CRC TME, we further dissected the gene expression profiles of the immune class and divided them into immune-activated and immune-suppressed subclasses based on a stromal-activated signature calculated using the NTP method. Interestingly, 46.9% of the patients in the immune class were assigned to the immune-activated subclasses, which showed lower enrichment of the stromal-related signatures and immunosuppressive cells—such as TGF-β, C-ECM, PD-1, Treg, and MDSC signatures—than that observed in the immune-suppressed subclass. This immune molecular classification was also validated in three independent cohorts. For example, the immune-activated subclass accounted for 40.5% of the patients, whereas the immune-suppressed subclass accounted for 59.5% of the patients in the immune class of the GSE17538 cohort. These results indicate that NMF may be used as a helpful classifier for stratifying immune molecular subtypes of patients with CRC.

Currently, treatment paradigms have been shifting from targeting the tumor cell compartment to focusing on the development of strategies for regulating the TME^54^. As a smaller number of patients respond to ICI therapy, identification of effective biomarkers for predicting patient suitability to immunotherapy is warranted. Recently, emerged immunological and non-invasive methods, such as liquid biopsy using peripheral T cell receptor repertoire of PD-1^+^ CD8^+^ lymphocytes, provide a non-invasive approach for selecting patients with metastatic CRC who could benefit from ICI treatment^55^. Meanwhile, with the advances in computational methods, such as SubMap and TIDE, a novel algorithm was established to predict ICI therapy response. SubMap analysis is conducted using an algorithm to evaluate the similarity in gene expression profiles between patients in different molecular subtypes and in patients with metastatic melanoma treated with ICIs^56^. Notably, here, the results of the SubMap analysis showed that patients in the immune-activated subtypes exhibited similar gene expression profiles to those of patients with melanoma who responded to anti-PD-1 immunotherapy, which suggested that patients in the immune-activated subclass could respond to PD-1. Moreover, survival analysis shows that patients in the immune class, especially in the immune-activated subclass, presented with better OS and DFS. These results may have clinical implications in terms of formulating prognosis and treatment decisions.

Genomic studies have revealed that CNAs, TMB, and neoantigens demonstrate a predictive value for ascertaining susceptibility to immunotherapies^57^. Lower CNAs were linked to the elevated infiltration of immunocytes and cytokines, which implied a higher likelihood of successful ICI therapy^58^. We analyzed the relationship between the established molecular subtypes and genetic features. We observed that CNA amplification was significantly lower in the immune class than that in the non-immune class. The levels of other canonical biomarkers that could help predict response to ICI, such as TIL and PD-1/PD-L1 expression, were significantly higher in the immune class, which confirmed patients in the immune class could potentially benefit from immunotherapy.

## Conclusions

Herein, we proposed a novel immune molecular classifier based on gene expression profiles analyzed using the NMF algorithm. Patients in the immune-activated subclass present with favorable prognosis and may respond positively to anti-PD-1 immunotherapy. This study provides new insights into tumor heterogeneity using an integrated and multifaceted approach to enhance the discovery of clinically important molecular subtypes that respond to immunotherapy.

## Supplementary Information

Supplementary Information.

## Data Availability

All data generated or analyzed during this study are included in this published article and its Supplementary Information files.

## References

[1] Siegel RL, Miller KD, Goding Sauer A, Fedewa SA, Butterly LF, Anderson JC, Cercek A, Smith RA, Jemal A (2020). Colorectal cancer statistics, 2020. CA Cancer J. Clin..

[2] Siegel RL, Miller KD, Fuchs HE, Jemal A (2021). Cancer statistics, 2021. CA Cancer J. Clin..

[3] Welch HG, Robertson DJ (2016). Colorectal cancer on the decline—Why screening can’t explain it all. N. Engl. J. Med..

[4] Keller DS, Berho M, Perez RO, Wexner SD, Chand M (2020). The multidisciplinary management of rectal cancer. Nat. Rev. Gastroenterol. Hepatol..

[5] Keum N, Giovannucci E (2019). Global burden of colorectal cancer: Emerging trends, risk factors and prevention strategies. Nat. Rev. Gastroenterol. Hepatol..

[6] Biller LH, Schrag D (2021). Diagnosis and treatment of metastatic colorectal cancer: A review. JAMA.

[7] Hull MA, Rees CJ, Sharp L, Koo S (2020). A risk-stratified approach to colorectal cancer prevention and diagnosis. Nat. Rev. Gastroenterol. Hepatol..

[8] Akimoto N, Ugai T, Zhong R, Hamada T, Fujiyoshi K, Giannakis M, Wu K, Cao Y, Ng K, Ogino S (2020). Rising incidence of early-onset colorectal cancer—A call to action. Nat. Rev. Clin. Oncol..

[9] Hofseth LJ, Hebert JR, Chanda A, Chen H, Love BL, Pena MM, Murphy EA, Sajish M, Sheth A, Buckhaults PJ (2020). Early-onset colorectal cancer: Initial clues and current views. Nat. Rev. Gastroenterol. Hepatol..

[10] Janney A, Powrie F, Mann EH (2020). Host-microbiota maladaptation in colorectal cancer. Nature.

[11] O'Keefe SJD (2016). Diet, microorganisms and their metabolites, and colon cancer. Nat. Rev. Gastroenterol. Hepatol..

[12] Ganesh K, Stadler ZK, Cercek A, Mendelsohn RB, Shia J, Segal NH, Diaz LA (2019). Immunotherapy in colorectal cancer: Rationale, challenges and potential. Nat. Rev. Gastroenterol. Hepatol..

[13] Hamid O, Robert C, Daud A, Hodi FS, Hwu WJ, Kefford R, Wolchok JD, Hersey P, Joseph RW, Weber JS (2013). Safety and tumor responses with lambrolizumab (anti-PD-1) in melanoma. N. Engl. J. Med..

[14] Topalian SL, Hodi FS, Brahmer JR, Gettinger SN, Smith DC, McDermott DF, Powderly JD, Carvajal RD, Sosman JA, Atkins MB (2012). Safety, activity, and immune correlates of anti-PD-1 antibody in cancer. N. Engl. J. Med..

[15] Topalian SL, Sznol M, McDermott DF, Kluger HM, Carvajal RD, Sharfman WH, Brahmer JR, Lawrence DP, Atkins MB, Powderly JD (2014). Survival, durable tumor remission, and long-term safety in patients with advanced melanoma receiving nivolumab. J. Clin. Oncol. Off. J. Am. Soc. Clin. Oncol..

[16] Binnewies M, Roberts EW, Kersten K, Chan V, Fearon DF, Merad M, Coussens LM, Gabrilovich DI, Ostrand-Rosenberg S, Hedrick CC (2018). Understanding the tumor immune microenvironment (TIME) for effective therapy. Nat. Med..

[17] Overman MJ, Lonardi S, Wong KYM, Lenz H-J, Gelsomino F, Aglietta M, Morse MA, Van Cutsem E, McDermott R, Hill A (2018). Durable clinical benefit with nivolumab plus ipilimumab in DNA mismatch repair-deficient/microsatellite instability-high metastatic colorectal cancer. J. Clin. Oncol. Off. J. Am. Soc. Clin. Oncol..

[18] Delaunay M, Cadranel J, Lusque A, Meyer N, Gounant V, Moro-Sibilot D, Michot J-M, Raimbourg J, Girard N, Guisier F (2017). Immune-checkpoint inhibitors associated with interstitial lung disease in cancer patients. Eur. Respir. J..

[19] Amaria RN, Reddy SM, Tawbi HA, Davies MA, Ross MI, Glitza IC, Cormier JN, Lewis C, Hwu W-J, Hanna E (2018). Neoadjuvant immune checkpoint blockade in high-risk resectable melanoma. Nat. Med..

[20] Motzer RJ, Tannir NM, McDermott DF, Arén Frontera O, Melichar B, Choueiri TK, Plimack ER, Barthélémy P, Porta C, George S (2018). Nivolumab plus ipilimumab versus sunitinib in advanced renal-cell carcinoma. N. Engl. J. Med..

[21] Le DT, Uram JN, Wang H, Bartlett BR, Kemberling H, Eyring AD, Skora AD, Luber BS, Azad NS, Laheru D (2015). PD-1 blockade in tumors with mismatch-repair deficiency. N. Engl. J. Med..

[22] Overman MJ, McDermott R, Leach JL, Lonardi S, Lenz H-J, Morse MA, Desai J, Hill A, Axelson M, Moss RA (2017). Nivolumab in patients with metastatic DNA mismatch repair-deficient or microsatellite instability-high colorectal cancer (CheckMate 142): An open-label, multicentre, phase 2 study. Lancet Oncol..

[23] Sadanandam A, Lyssiotis CA, Homicsko K, Collisson EA, Gibb WJ, Wullschleger S, Ostos LC, Lannon WA, Grotzinger C, Del Rio M (2013). A colorectal cancer classification system that associates cellular phenotype and responses to therapy. Nat. Med..

[24] Hargadon KM, Johnson CE, Williams CJ (2018). Immune checkpoint blockade therapy for cancer: An overview of FDA-approved immune checkpoint inhibitors. Int. Immunopharmacol..

[25] Lavin Y, Kobayashi S, Leader A, Amir ED, Elefant N, Bigenwald C, Remark R, Sweeney R, Becker CD, Levine JH (2017). Innate immune landscape in early lung adenocarcinoma by paired single-cell analyses. Cell.

[26] Papadopoulos KP, Gluck L, Martin LP, Olszanski AJ, Tolcher AW, Ngarmchamnanrith G, Rasmussen E, Amore BM, Nagorsen D, Hill JS (2017). First-in-human study of AMG 820, a monoclonal anti-colony-stimulating factor 1 receptor antibody, in patients with advanced solid tumors. Clin. Cancer Res. Off. J. Am. Assoc. Cancer Res..

[27] Zhao Y, Wang H, Pei J (2021). Deep non-negative matrix factorization architecture based on underlying basis images learning. IEEE Trans. Pattern Anal. Mach. Intell..

[28] Anderson A, Douglas PK, Kerr WT, Haynes VS, Yuille AL, Xie J, Wu YN, Brown JA, Cohen MS (2014). Non-negative matrix factorization of multimodal MRI, fMRI and phenotypic data reveals differential changes in default mode subnetworks in ADHD. Neuroimage.

[29] Ma Y, He T, Jiang X (2019). Projection-based neighborhood non-negative matrix factorization for lncRNA–protein interaction prediction. Front. Genet..

[30] Sia D, Jiao Y, Martinez-Quetglas I, Kuchuk O, Villacorta-Martin C, Castro de Moura M, Putra J, Camprecios G, Bassaganyas L, Akers N (2017). Identification of an immune-specific class of hepatocellular carcinoma, based on molecular features. Gastroenterology.

[31] Meng J, Zhou Y, Lu X, Bian Z, Chen Y, Zhou J, Zhang L, Hao Z, Zhang M, Liang C (2020). Immune response drives outcomes in prostate cancer: Implications for immunotherapy. Mol. Oncol..

[32] Tan Q, Huang Y, Deng K, Lu M, Wang L, Rong Z, Zhao W, Li S, Xu Z, Fan L (2020). Identification immunophenotyping of lung adenocarcinomas based on the tumor microenvironment. J. Cell. Biochem..

[33] Yang C, Huang X, Liu Z, Qin W, Wang C (2020). Metabolism-associated molecular classification of hepatocellular carcinoma. Mol. Oncol..

[34] Rooney MS, Shukla SA, Wu CJ, Getz G, Hacohen N (2015). Molecular and genetic properties of tumors associated with local immune cytolytic activity. Cell.

[35] Saltz J, Gupta R, Hou L, Kurc T, Singh P, Nguyen V, Samaras D, Shroyer KR, Zhao T, Batiste R (2018). Spatial organization and molecular correlation of tumor-infiltrating lymphocytes using deep learning on pathology images. Cell Rep..

[36] Yoshihara K, Shahmoradgoli M, Martínez E, Vegesna R, Kim H, Torres-Garcia W, Treviño V, Shen H, Laird PW, Levine DA (2013). Inferring tumour purity and stromal and immune cell admixture from expression data. Nat. Commun..

[37] Thorsson V, Gibbs DL, Brown SD, Wolf D, Bortone DS, Ou Yang T-H, Porta-Pardo E, Gao GF, Plaisier CL, Eddy JA (2018). The immune landscape of cancer. Immunity.

[38] Guinney J, Dienstmann R, Wang X, de Reyniès A, Schlicker A, Soneson C, Marisa L, Roepman P, Nyamundanda G, Angelino P (2015). The consensus molecular subtypes of colorectal cancer. Nat. Med..

[39] Dienstmann R, Vermeulen L, Guinney J, Kopetz S, Tejpar S, Tabernero J (2017). Consensus molecular subtypes and the evolution of precision medicine in colorectal cancer. Nat. Rev. Cancer.

[40] Moffitt RA, Marayati R, Flate EL, Volmar KE, Loeza SGH, Hoadley KA, Rashid NU, Williams LA, Eaton SC, Chung AH (2015). Virtual microdissection identifies distinct tumor- and stroma-specific subtypes of pancreatic ductal adenocarcinoma. Nat. Genet..

[41] Batlle E, Massagué J (2019). Transforming growth factor-β signaling in immunity and cancer. Immunity.

[42] Chakravarthy A, Khan L, Bensler NP, Bose P, De Carvalho DD (2018). TGF-β-associated extracellular matrix genes link cancer-associated fibroblasts to immune evasion and immunotherapy failure. Nat. Commun..

[43] Groth C, Hu X, Weber R, Fleming V, Altevogt P, Utikal J, Umansky V (2019). Immunosuppression mediated by myeloid-derived suppressor cells (MDSCs) during tumour progression. Br. J. Cancer.

[44] Monteran L, Erez N (1835). The dark side of fibroblasts: Cancer-associated fibroblasts as mediators of immunosuppression in the tumor microenvironment. Front. Immunol..

[45] Jiang P, Gu S, Pan D, Fu J, Sahu A, Hu X, Li Z, Traugh N, Bu X, Li B (2018). Signatures of T cell dysfunction and exclusion predict cancer immunotherapy response. Nat. Med..

[46] Bassaganyas L, Pinyol R, Esteban-Fabró R, Torrens L, Torrecilla S, Willoughby CE, Franch-Expósito S, Vila-Casadesús M, Salaverria I, Montal R (2020). Copy-number alteration burden differentially impacts immune profiles and molecular features of hepatocellular carcinoma. Clin. Cancer Res. Off. J. Am. Assoc. Cancer Res..

[47] Miao D, Margolis CA, Vokes NI, Liu D, Taylor-Weiner A, Wankowicz SM, Adeegbe D, Keliher D, Schilling B, Tracy A (2018). Genomic correlates of response to immune checkpoint blockade in microsatellite-stable solid tumors. Nat. Genet..

[48] Malta TM, Sokolov A, Gentles AJ, Burzykowski T, Poisson L, Weinstein JN, Kamińska B, Huelsken J, Omberg L, Gevaert O (2018). Machine learning identifies stemness features associated with oncogenic dedifferentiation. Cell.

[49] Wu T, Dai Y (2017). Tumor microenvironment and therapeutic response. Cancer Lett..

[50] Devarajan K (2008). Nonnegative matrix factorization: An analytical and interpretive tool in computational biology. PLoS Comput. Biol..

[51] Meng J, Lu X, Zhou Y, Zhang M, Ge Q, Zhou J, Hao Z, Gao S, Yan F, Liang C (2021). Tumor immune microenvironment-based classifications of bladder cancer for enhancing the response rate of immunotherapy. Mol. Ther. Oncolytics.

[52] Calon A, Espinet E, Palomo-Ponce S, Tauriello DVF, Iglesias M, Céspedes MV, Sevillano M, Nadal C, Jung P, Zhang XHF (2012). Dependency of colorectal cancer on a TGF-β-driven program in stromal cells for metastasis initiation. Cancer Cell.

[53] Karakasheva TA, Lin EW, Tang Q, Qiao E, Waldron TJ, Soni M, Klein-Szanto AJ, Sahu V, Basu D, Ohashi S (2018). IL-6 mediates cross-talk between tumor cells and activated fibroblasts in the tumor microenvironment. Cancer Res..

[54] Hinshaw DC, Shevde LA (2019). The tumor microenvironment innately modulates cancer progression. Cancer Res..

[55] Russano M, Napolitano A, Ribelli G, Iuliani M, Simonetti S, Citarella F, Pantano F, Dell'Aquila E, Anesi C, Silvestris N (2020). Liquid biopsy and tumor heterogeneity in metastatic solid tumors: The potentiality of blood samples. J. Exp. Clin. Cancer Res. CR.

[56] Hoshida Y, Brunet J-P, Tamayo P, Golub TR, Mesirov JP (2007). Subclass mapping: Identifying common subtypes in independent disease data sets. PLoS ONE.

[57] McGranahan N, Furness AJS, Rosenthal R, Ramskov S, Lyngaa R, Saini SK, Jamal-Hanjani M, Wilson GA, Birkbak NJ, Hiley CT (2016). Clonal neoantigens elicit T cell immunoreactivity and sensitivity to immune checkpoint blockade. Science (New York, NY).

[58] Keenan TE, Burke KP, Van Allen EM (2019). Genomic correlates of response to immune checkpoint blockade. Nat. Med..

