# Supplementary material for: Identification of immune-related subtypes of colorectal cancer to improve antitumor immunotherapy
Source: Sci Rep. 2021 Sep 30;11:19432. doi: 10.1038/s41598-021-98966-x (PMC8484460; doi:10.1038/s41598-021-98966-x)
Supplement: Supplementary file 1 — Supplementary Information. [file 41598_2021_98966_MOESM1_ESM.docx]

Identification of Immune-Related Subtypes of Colorectal Cancer to Improve Antitumor Immunotherapy

Xiaobo Zheng ^1^, Yong Gao ^2^, Chune Yu ^3^, Guiquan Fan ^4^, Pengwu Li ^5^, Ming Zhang ^1,6^, Jing Yu ^3,^*, Mingqing Xu ^1,7,^*

^1^ Department of Liver Surgery, West China Hospital, Sichuan University, Chengdu, Sichuan 610041, China

^2^ Department of Gastroenterology, Second Affiliated Hospital, Army Medical University, Chongqing 400037, China

^3^ Laboratory of Tumor Targeted and Immune Therapy, Clinical Research Center for Breast, State Key Laboratory of Biotherapy, West China Hospital, Sichuan University, Chengdu, Sichuan 610041, China

^4^ Department of General Surgery, First People's Hospital of Liangshan Yi Autonomous Prefecture, Liangshan, Sichuan 615000, China

^5^ Department of Hepatobiliary Surgery, Chongzhou People's Hospital, Chengdu, Sichuan 611200, China

^6^ Department of General Surgery, Mianzhu Hospital of West China hospital, Sichuan University, Mianzhu, Sichuan 618200, China

^7^ Department of Hepatopancreatobiliary Surgery, Meishan City People's Hospital, Meishan Hospital of West China Hospital, Sichuan University, Meishan, Sichuan 610041, China

* Correspondence: yj20110312@163.com (J.Y.); [xumingqing0018@163.com](mailto:xumingqing0018@163.com) (M.X.)

**
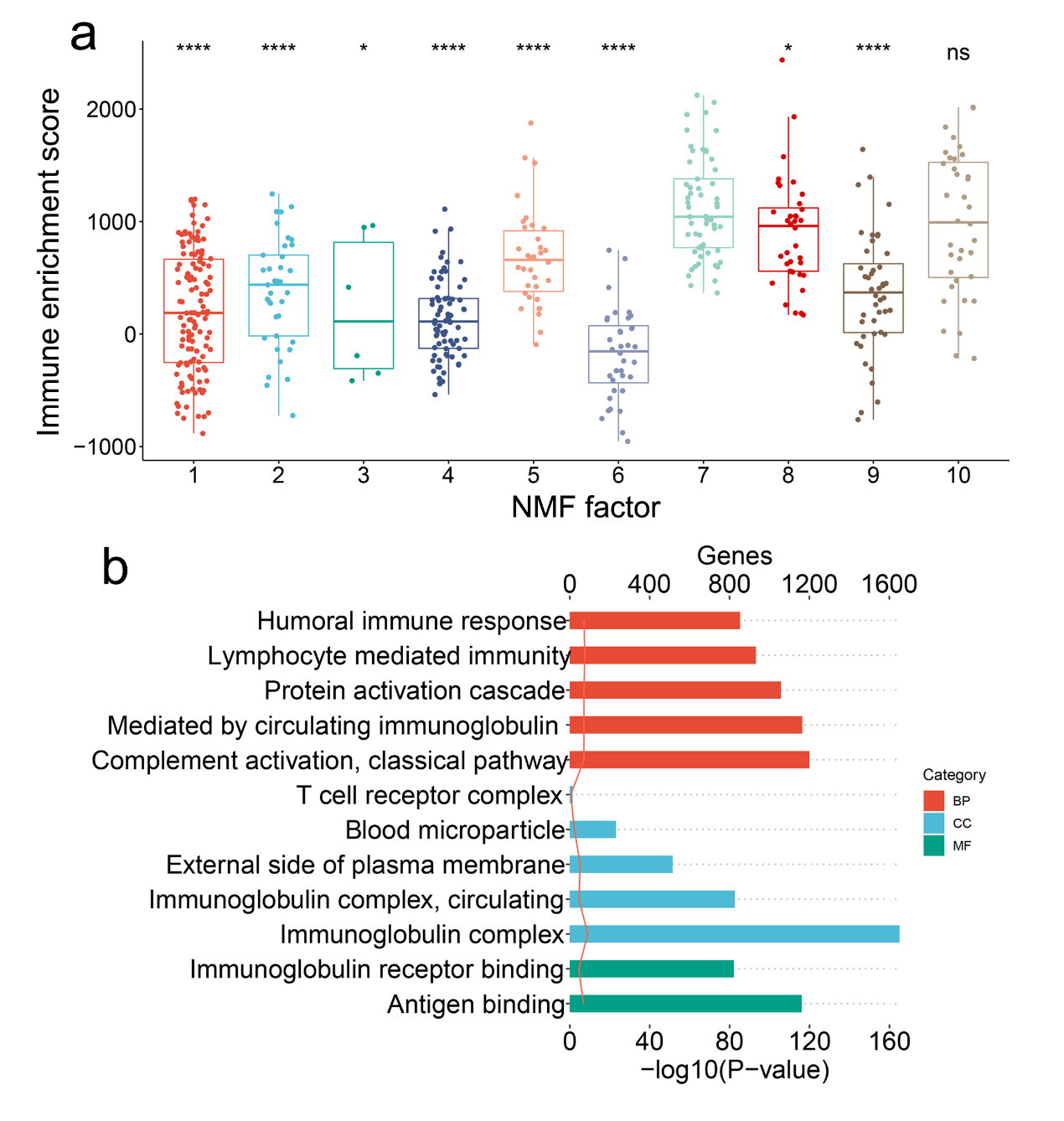
Supplementary Figure S1. Immune enrichment score of NMF factors and GO enrichment of top 150 exemplar genes.** (**a**) Immune enrichment score among ten NMF factors generated by using the ESTIMATE algorithm. ns, p > 0.05; *, p < 0.05; **, p < 0.01; ***, p < 0.001; ****, p < 0.0001; the Student’s *t*-test, compared with factor 7. (**b**) GO enrichment of top 150 exemplar genes of the immune factor. BP, biological process; CC, cellular component; MF, molecular function.

**
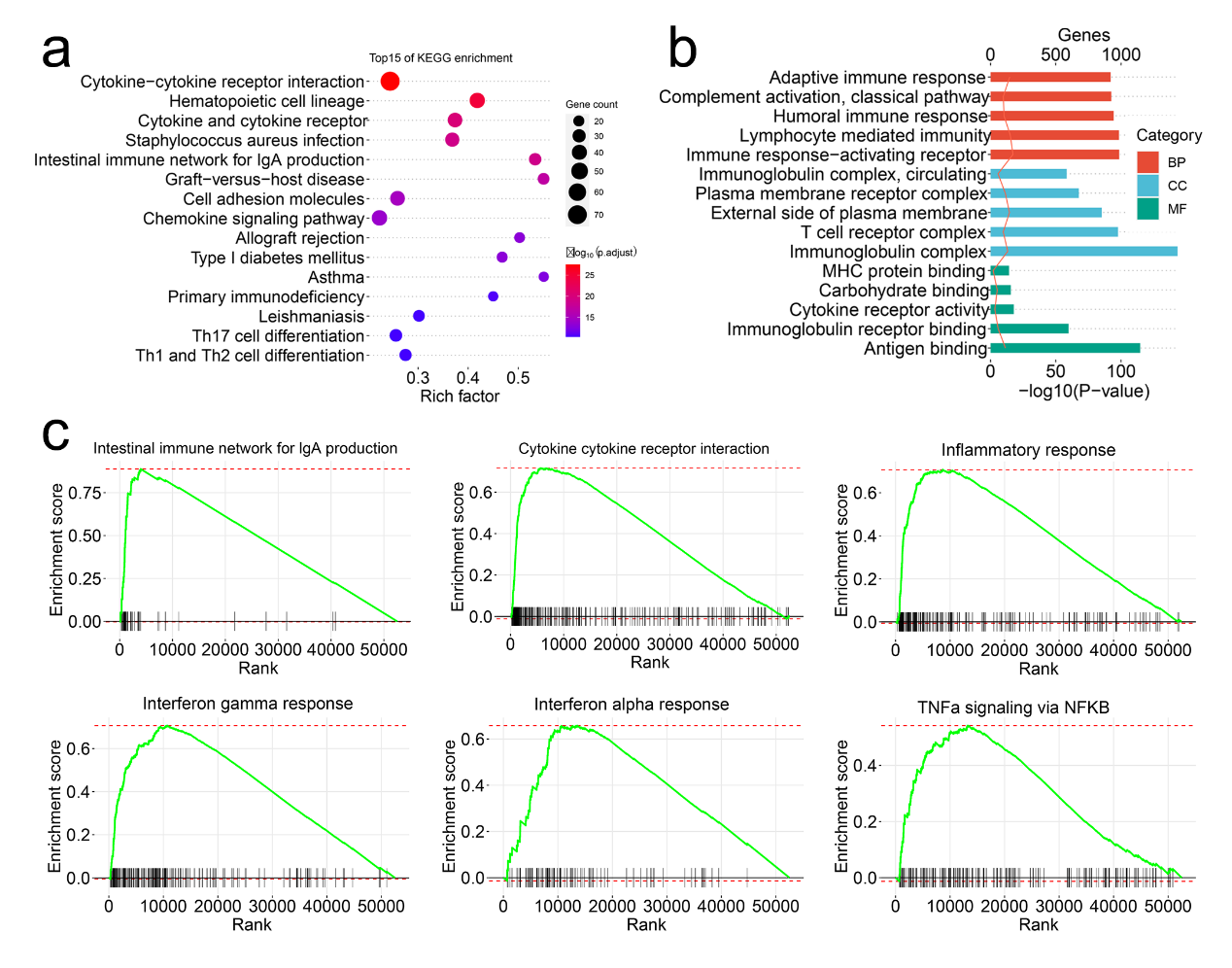
Supplementary Figure S2. Gene set enrichment of DEGs between immune and non-immune classes.** (**a**) KEGG enrichment of DEGs between immune and non-immune classes. (**b**) GO enrichment of DEGs between immune and non-immune classes. (**c**) GSEA enrichment of all genes between immune and non-immune classes. BP, biological process; CC, cellular component; MF, molecular function.

**
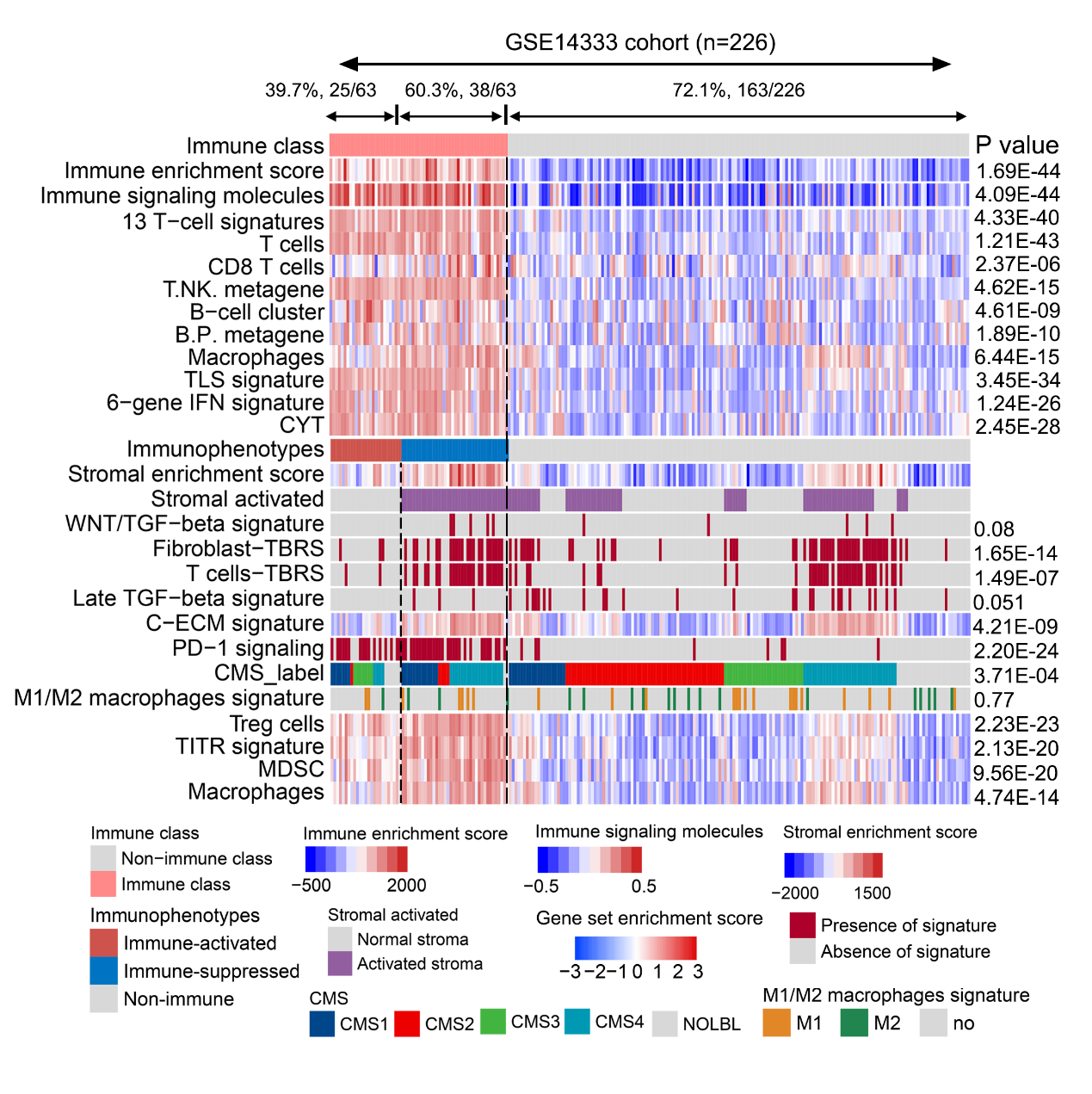
Supplementary Figure S3. Validation of the immunophenotypes in the GSE14333 cohort.**

**
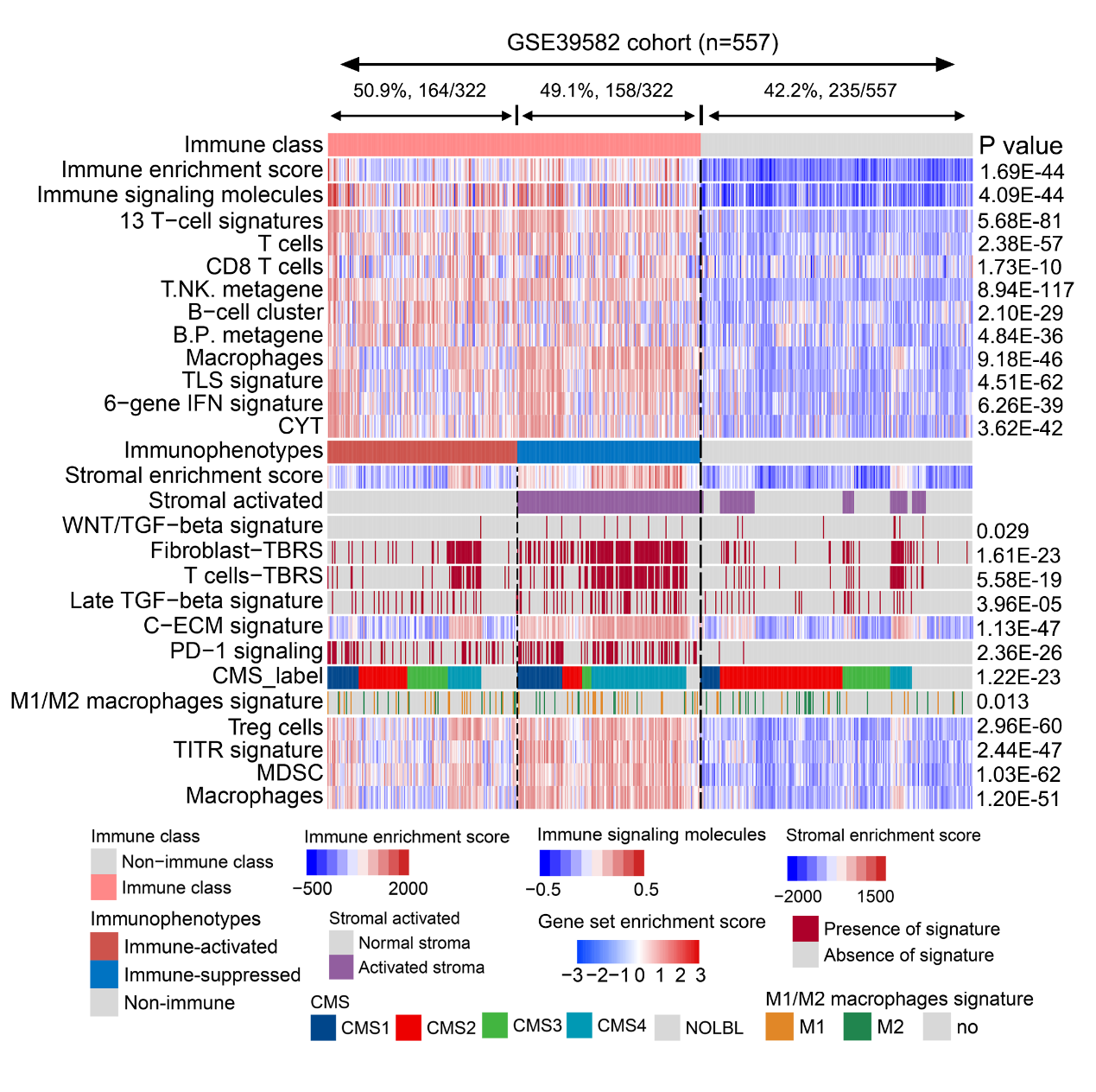
Supplementary Figure S4. Validation of the immunophenotypes in the GSE39582 cohort.**

**
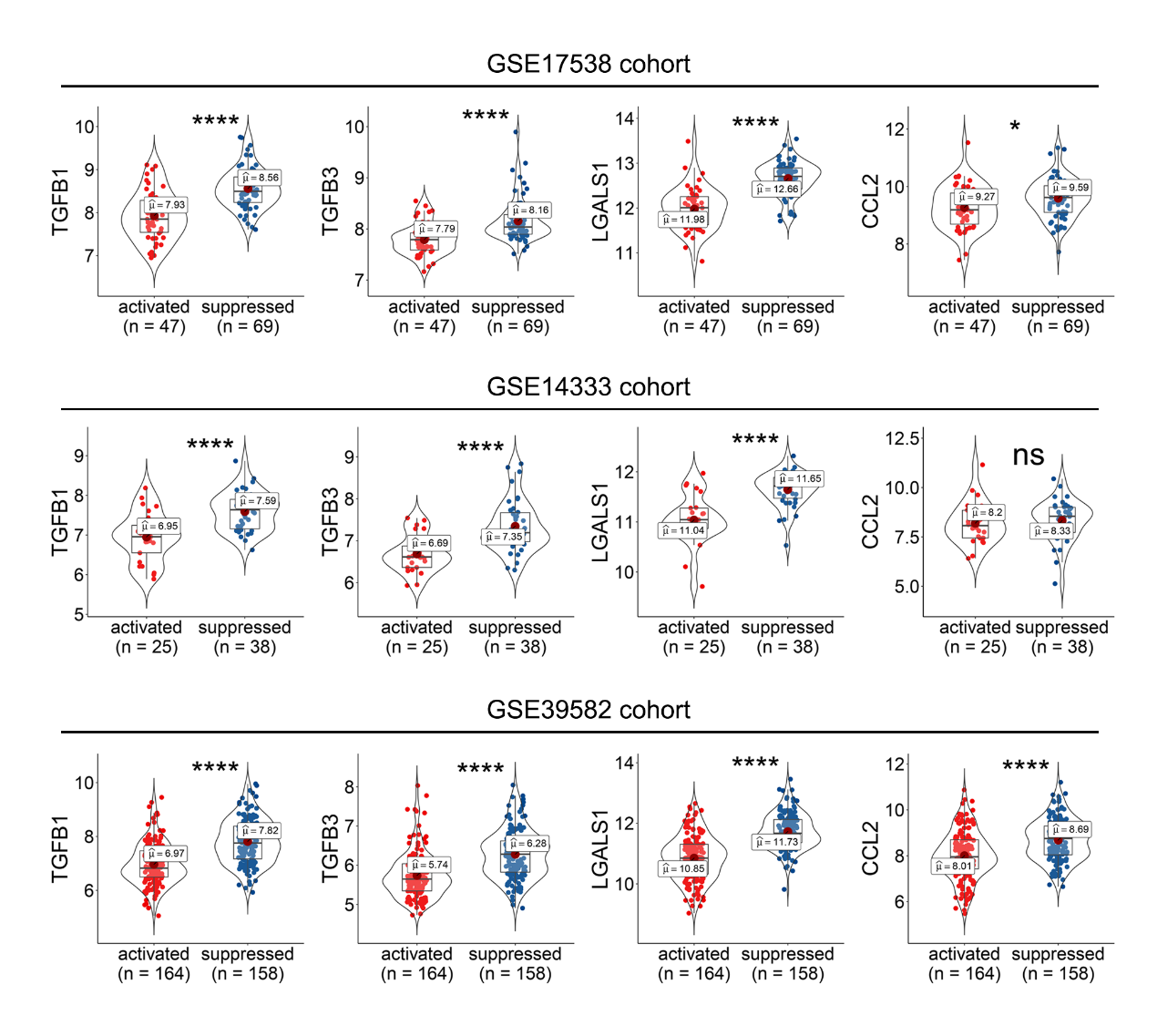
Supplementary Figure S5. Expression levels of genes related to immune suppression between the immune-activated and immune-suppressed subclasses in the three validation cohorts.** ns, p > 0.05; *, p < 0.05; **, p < 0.01; ***, p < 0.001; ****, p < 0.0001; the Student’s *t*-test.

**
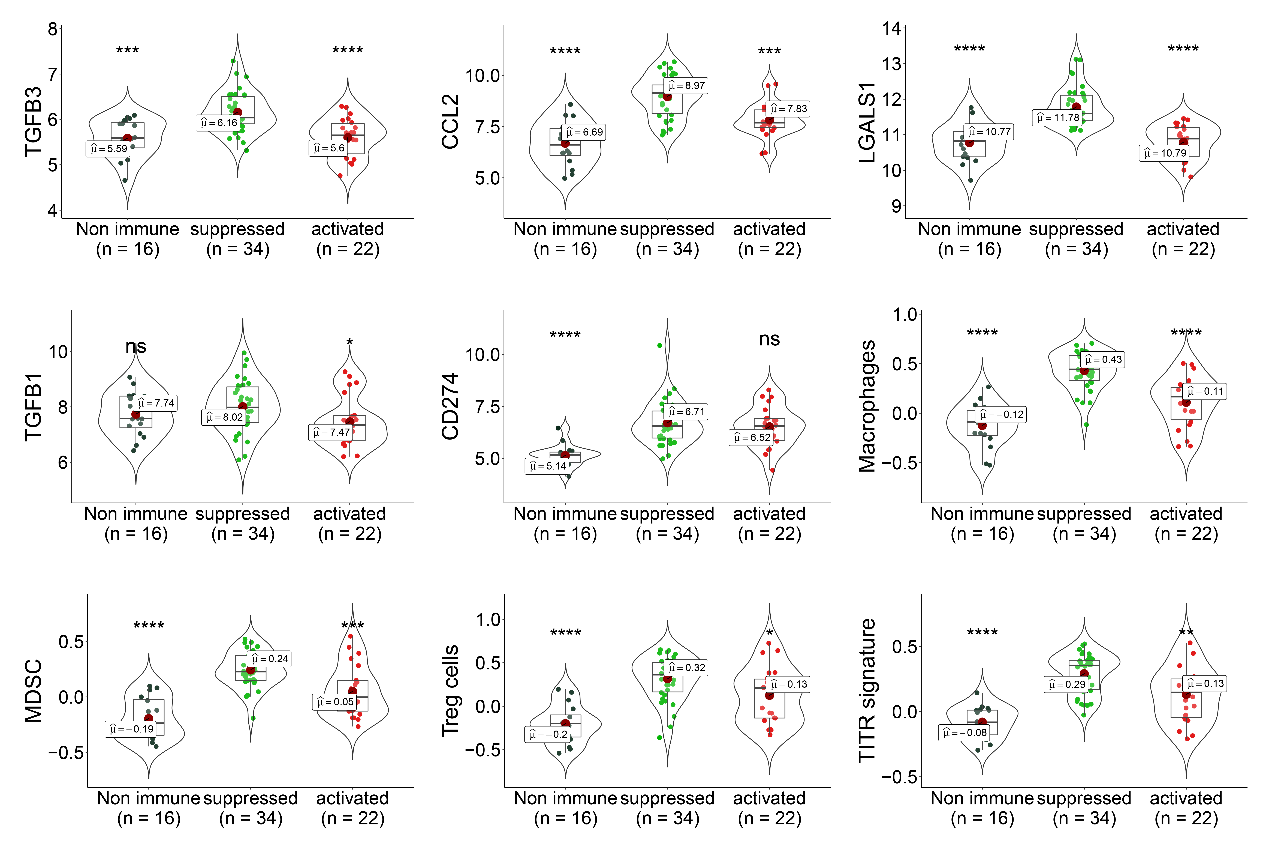
**

**Supplementary Figure S6. Expression levels of signalings and genes related to immune suppression between the immunophenotypes of MSI CRC patients in the GSE39582 cohorts.** ns, p > 0.05; *, p < 0.05; **, p < 0.01; ***, p < 0.001; ****, p < 0.0001; the Student’s *t*-test, compared with immune-suppressed group.

**
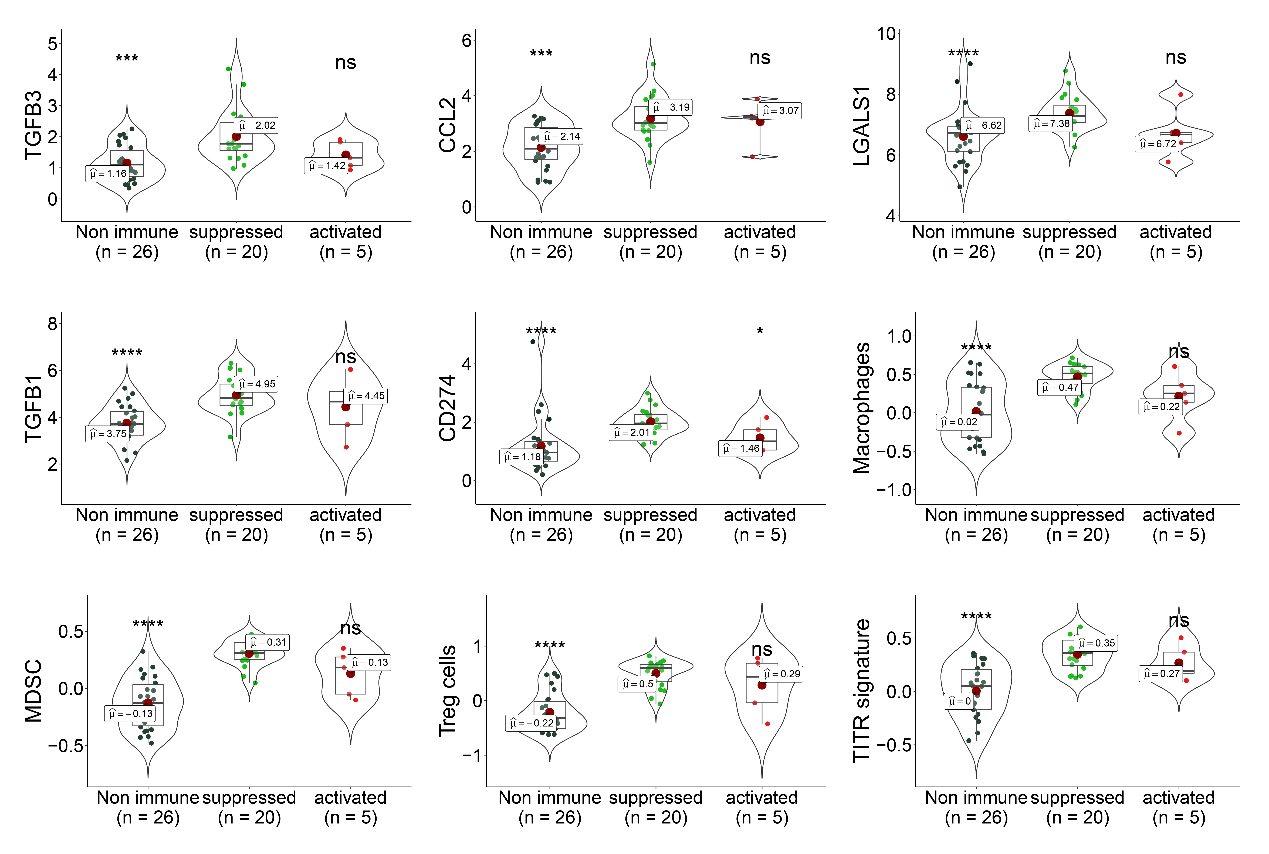
**

**Supplementary Figure S7. Expression levels of signalings and genes related to immune suppression between the immunophenotypes of MSI CRC patients in the TCGA cohorts.** ns, p > 0.05; *, p < 0.05; **, p < 0.01; ***, p < 0.001; ****, p < 0.0001; the Student’s *t*-test, compared with immune-suppressed group.

**Supplementary Table S1. Immune-associated gene signatures used in this study.**

| **Signature Name** | **Reference** |
| --- | --- |
| Immune enrichment score | Yoshihara *et al.* Nat Commun. 2013;4:2612 |
| Stromal enrichment score | Yoshihara *et al.* Nat Commun. 2013;4:2612 |
| Immune signaling molecules | Cancer Genome Atlas Network. Cell. 2015;161:1681-96 |
| 13 T-cell signature | Spranger *et al.* Proc Natl AcadSci U S A. 2016; 113 (48):E7759-E7768. |
| T cells | Bindea *et al.* Immunity. 2013;39:782-95 |
| CD8 T cells | Bindea *et al.* Immunity. 2013;39:782-95 |
| Treg cells | Angelova *et al.* Genome Biol. 2015;16:64 |
| TITR signature | Magnuson *et al.* Proceedings of the National Academy of Sciences of the United States of America. 2018;115(45):E10672-e81 |
| MDSC | Angelova *et al.* Genome Biol. 2015;16:64 |
| T.NK. metagene | Alistar *et al.* Genome Med. 2014;6:80 |
| B-cell cluster | Iglesia *et al.* Clin Cancer Res. 2014; 20 (14):3818–3829. |
| B.P. metagene | Alistar *et al.* Genome Med. 2014;6:80 |
| Macrophages | Bindea *et al.* Immunity. 2013;39:782-95 |
| TLS signature | Finkin *et al.* Nat Immunol. 2015;16:1235-44 |
| 6-gene IFN signature | Chow *et al.* J Clin Oncol. 34, (suppl; abstr 6010) 2016 |
| CYT | Rooney *et al.* Cell. 2015;160:48-61 |
| WNT/TGF-β signature | Lachenmayer *et al.* Clin Cancer Res. 2012;18:4997-5007 |
| C-ECM signature | Chakravarthy *et al.* Nature Communications. 2018;9(1) |
| M1/M2 macrophages | Coates PJ, *et al*. Cancer Res 2008;68:450-6 |
| Late TGFB signature | Coulouarn C, *et al*. Hepatology. 2008 Jun;47(6):2059-67 |
| T cells-TBRS | Calon A, *et al*. Cancer Cell 2012;22:571-84 |
| Fibroblast-TBRS | Calon A, *et al*. Cancer Cell 2012;22:571-84 |
| Stromal activated | Moffitt RA, *et al*. Nat Genet 2015;47:1168-78 |
| PD-1 signaling | Quigley M, *et al*. Nat Med 2010;16:1147-51 |
| CMS label | Guinney, *et al*. Nat Med 21, 1350–1356 (2015). |
| IS | Thorsson V, *et al*. Immunity 2018;48(4):812e830.e14. |

TITR, tumor-infiltrating Tregs; MDSC, myeloid-derived suppressor cell; IFN, interferon; TLS, tertiary lymphoid structure; CYT, cytolytic activity score; C-ECM, cancer-associated extracellular matrix.

**Supplementary Table S2. Univariate and multivariate analysis of risk factors of survival**

|  |  | OS | | DFS | |
| --- | --- | --- | --- | --- | --- |
|  | NO. | Hazard rations (95%CI) | P-value | Hazard rations (95%CI) | P-value |
| **Univariate analysis** |  |  |  |  |  |
| Age |  |  |  |  |  |
| <70 | 274 |  |  |  |  |
| >=70 | 214 | 2.793(1.732-4.503) | 2.5e-05 *** | 1.342(0.8582-2.099) | 0.197 |
| Gender |  |  |  |  |  |
| Female | 221 |  |  |  |  |
| Male | 267 | 1.168(0.7339-1.858) | 0.513 | 1.428(0.897-2.273) | 0.133 |
| TNM stage |  |  |  |  |  |
| Stage I-II | 264 |  |  |  |  |
| Stage III-IV | 206 | 2.98(1.774-5.004) | 3.66e-05 *** | 2.583(1.619-4.12) | 6.79e-05 *** |
| Tumor stage |  |  |  |  |  |
| T1-2 | 107 |  |  |  |  |
| T3-T4 | 379 | 1.646(0.7857-3.446) | 0.187 | 3.474(1.402-8.608) | 0.00714 ** |
| Lymph node metastasis |  |  |  |  |  |
| N0 | 280 |  |  |  |  |
| N1-N2 | 205 | 2.945(1.809-4.792) | 1.38e-05 *** | 2.613(1.662-4.111) | 3.22e-05 *** |
| Distant metastasis |  |  |  |  |  |
| M0 | 361 |  |  |  |  |
| M1 | 73 | 3.133(1.801-5.449) | 5.27e-05 *** | 3.959(2.312-6.779) | 5.34e-07 *** |
| Tumor size |  |  |  |  |  |
|  | 488 | 1.826(1.143-2.917) | 0.0117 * | 1.128(0.7145-1.78) | 0.606 |
| Immunephenotypes |  |  |  |  |  |
| Immune suppressed | 111 |  |  |  |  |
| Non immune | 279 | 0.6298(0.36319-1.0920) | 0.09965 | 0.7345(0.4140-1.3030) | 0.29143 |
| Immune activated | 98 | 0.2628(0.09666-0.7143) | 0.00881 ** | 0.2715(0.1047-0.7039) | 0.00731 ** |
| **Multivariate analysis** |  |  |  |  |  |
| Age |  |  |  |  |  |
| <70 | 274 |  |  |  |  |
| >=70 | 214 | 5.656(2.68176-11.9304) | 5.35e-06 *** |  |  |
| TNM stage |  |  |  |  |  |
| Stage I-II | 264 |  |  |  |  |
| Stage III-IV | 206 | 6.734e-07(0.00000-Inf) | 0.9973 | 0.7481(0.08881-6.3015) | 0.78952 |
| Tumor stage |  |  |  |  |  |
| T1-2 |  |  |  |  |  |
| T3-T4 |  |  |  | 1.7455(0.66335-4.5929) | 0.25913 |
| Lymph node metastasis |  |  |  |  |  |
| N0 | 280 |  |  |  |  |
| N1-N2 | 205 | 5.358e+06(0.00000-Inf) | 0.9971 | 1.6941(0.22318-12.8599) | 0.61023 |
| Distant metastasis |  |  |  |  |  |
| M0 | 361 |  |  |  |  |
| M1 | 73 | 1.393(0.57449-3.3789) | 0.4631 | 3.1632(1.55738-6.4247) | 0.00145 ** |
| Tumor size |  |  |  |  |  |
|  | 488 | 1.971(1.14535-3.3905) | 0.0143 * |  |  |
| Immunephenotypes |  |  |  |  |  |
| Immune-suppressed | 111 |  |  |  |  |
| Non-immune | 279 | 4.752e-01(0.23117-0.9768) | 0.0430 * | 0.6728(0.36382-1.2442) | 0.20643 |
| Immune-activated | 98 | 2.219e-01(0.06826-0.7212) | 0.0123 * | 0.3440(0.11986-0.9873) | 0.04729 * |

*, p < 0.05; **, p < 0.01; ***, p < 0.001

**Supplementary Table S3. Correlation between immune-related subtypes and clinicopathological parameters**

|  | **Non-immune** | **Immune-suppressed** | **Immune-activated** | **P value** |
| --- | --- | --- | --- | --- |
| **Age** |  |  |  | 0.0978 |
|  | 51.59-77.89 | 55.82-79.09 | 56.36-79.54 |  |
| **Gender** |  |  |  | 0.635 |
| Female | 131 | 46 | 44 |  |
| Male | 148 | 65 | 54 |  |
| **pTNM:M** |  |  |  | 0.2384 |
| M1 | 47 | 15 | 11 |  |
| **pTNM:N** |  |  |  | 0.342 |
| N0 | 152 | 67 | 61 |  |
| N1 | 74 | 20 | 20 |  |
| N2 | 50 | 24 | 17 |  |
| **pTNM:T** |  |  |  | 0.7049 |
| T1 | 8 | 3 | 6 |  |
| T2 | 50 | 20 | 20 |  |
| T3 | 189 | 76 | 64 |  |
| T4 | 31 | 12 | 7 |  |
| **pTNM** |  |  |  | 0.4143 |
| Stage I | 44 | 20 | 25 |  |
| Stage II | 97 | 46 | 32 |  |
| Stage III | 77 | 28 | 27 |  |
| Stage IV | 47 | 15 | 12 |  |
| **CIMP** |  |  |  | 0.3582 |
| CIMP-high | 28 | 20 | 8 |  |
| CIMP-low | 39 | 17 | 19 |  |
| CIMP-negative | 133 | 62 | 55 |  |
| **Kras mutation** |  |  |  | 0.054 |
| Mutation | 73 | 19 | 12 |  |
| No Mutation | 104 | 50 | 33 |  |
| **Braf mutation** |  |  |  | 0.4593 |
| Mutation | 11 | 7 | 4 |  |
| No Mutation | 166 | 62 | 41 |  |
| **Longest dimension of samples** |  |  |  | 0.4113 |
|  | 0.72-1.85 | 0.73-1.8 | 0.7-1.68 |  |

DFS, disease-free survival; OS, overall survival; CIMP, CpG island methylator phenotype.
